# Recent advances in irradiation-mediated synthesis and tailoring of inorganic nanomaterials for photo-/electrocatalysis

**DOI:** 10.1039/d4na00806e

**Published:** 2024-11-27

**Authors:** Shoushuang Huang, Can Yue, Kajsa Uvdal, Zhangjun Hu

**Affiliations:** a School of Environmental and Chemical Engineering, Shanghai University Shanghai 200444 China sshuang@shu.edu.cn; b Division of Molecular Surface Physics & Nanoscience, Department of Physics, Chemistry and Biology, Linköping University Linköping 58183 Sweden zhangjun.hu@liu.se

## Abstract

Photo-/electrocatalysis serves as a cornerstone in addressing global energy shortages and environmental pollution, where the development of efficient and stable catalysts is essential yet challenging. Despite extensive efforts, it's still a formidable task to develop catalysts with excellent catalytic behaviours, stability, and low cost. Because of its high precision, favorable controllability and repeatability, radiation technology has emerged as a potent and versatile strategy for the synthesis and modification of nanomaterials. Through meticulous control of irradiation parameters, including energy, fluence and ion species, various inorganic photo-/electrocatalysts can be effectively synthesized with tailored properties. It also enables the efficient adjustment of physicochemical characteristics, such as heteroatom-doping, defect generation, heterostructure construction, micro/nanostructure control, and so on, all of which are beneficial for lowering reaction energy barriers and enhancing energy conversion efficiency. This review comprehensively outlines the principles governing radiation effects on inorganic catalysts, followed by an in-depth discussion of recent advancements in irradiation-enhanced catalysts for various photo-/electrocatalytic applications, such as hydrogen and oxygen evolution reactions, oxygen reduction reactions, and photocatalytic applications. Furthermore, the challenges associated with ionizing and non-ionizing radiation are discussed and potential avenues for future development are outlined. By summarizing and articulating these innovative strategies, we aim to inspire further development of sustainable energy and environmental solutions to drive a greener future.

## Abstract 2

In this review, the mechanisms and applications of radiation technology in the development of inorganic photo-/electrocatalysts are summarized, and its recent research progress, challenges, and future development prospects are further explored.

## Graphical abstract

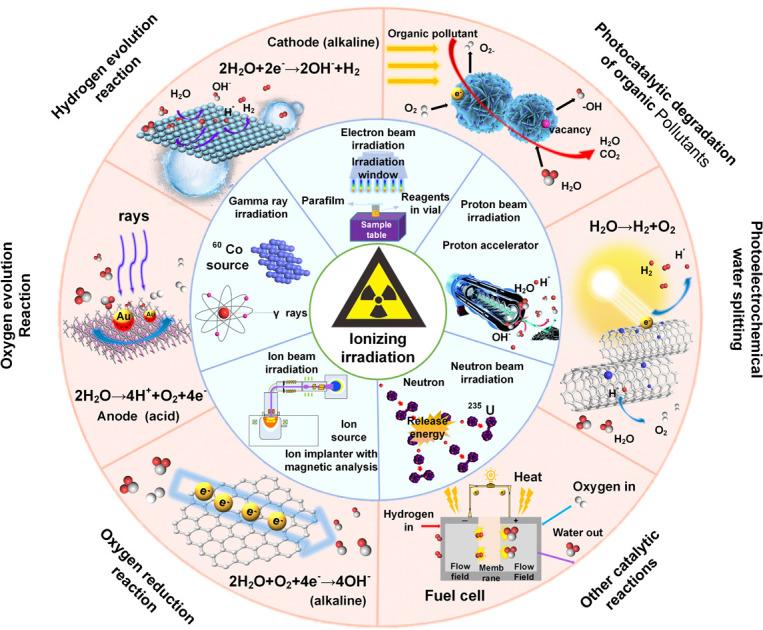

## 1 Introduction

In recent years, photocatalysis and electrocatalysis have shown great advantages and broad application prospects in coping with the challenges of renewable clean energy.^1^ Photocatalysis, driven by solar energy, initiates photocatalytic reactions *via* the generation of photo-induced electrons and holes, providing a friendly and sustainable way to transform solar energy into chemical energy (*e.g.* H_2_ production through water splitting), which is beneficial for tackling environmental challenges.^2,3^ Electrocatalysis, on the other hand, can realize efficient energy conversion through electrocatalytic reactions on well-designed electrodes, such as hydrogen evolution reaction (HER), oxygen evolution reaction (OER), oxygen reduction reaction (ORR), and so on, opening up broad prospects for the development of efficient energy devices with excellent performances.^4–8^ In this regard, photocatalysis and electrocatalysis are of great significance for achieving green sustainable development.^9^ However, the efficiency, selectivity, and stability of photo-/electro-catalytic reactions strongly depend on the utilized nanomaterials. For example, the band gap structure of a photocatalyst directly affects its light absorption and separation efficiency for photo-induced electrons and holes, which subsequently determines its photocatalytic performance.^10,11^ As a result, the controllable preparation and modification of photo-/electrocatalysts to achieve excellent catalytic activity, selectivity, and stability is essential for advancing catalytic technologies.

Currently, there are many ways to fabricate inorganic catalysts with different morphologies, phases and chemical constitutions, mainly including the top down and the bottom-up methods.^12–15^ However, certain inherent limitations in these methods may restrict their broader practical applications across different contexts. In particular, the “top-down” methods often suffer from inconsistencies in the size of particles, probable contamination from the starting bulk raw materials, and lack of ability to achieve perfectly homogeneous chemical compositions, which brings about their reduced effectiveness in all applications where a high degree of accuracy or uniformity is required. On the other hand, while giving much better control over the size and shape of the catalysts, the bottom-up methods have several drawbacks: complex synthesis processes mostly involving multiple steps, high sensitivity to conditions, variable yields, and difficulties in reproducibility, which are critical constraints when it comes to scaling up production.^16^ Moreover, both “top-down” and “bottom-up” processes may use toxic and expensive chemicals or harsh conditions, posing environmental and safety concerns. These disadvantages highlight the urgent need for developing new methods to fabricate excellent photo/electro-catalysts.

**Fig. 1:**
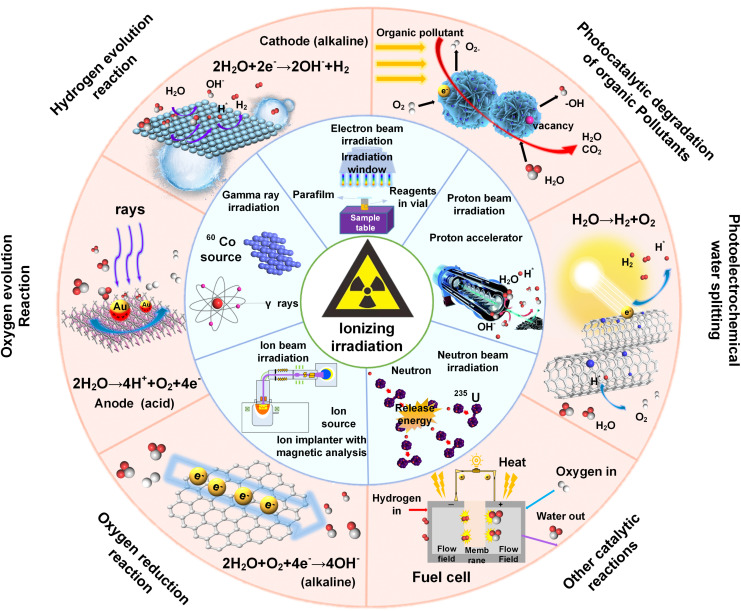
Schematic illustration of the application of ionizing irradiation technology in photo-/electrocatalysis.

Recently, the use of radiation technology to control the synthesis and modification of nano-catalysts has received extensive attention.^17^ Radiation is the emission or transmission of energy *via* electromagnetic waves or particle beams. The radiation source can be regarded as an energy reservoir, and the energy is released in the form of photons, particles, and other basic carriers. These carriers can transfer energy to matter through direct interactions with atoms and/or molecules, resulting in various unique radiation effects, which are suitable for developing multi-functional nanomaterials for energy storage and conversion (Fig. 1). Recently, several reviews have summarized the use of ion irradiation in the fields of textiles, supercapacitors, microelectronics, and solar cells.^10,18–22^ However, a comprehensive review encompassing the full spectrum of electromagnetic waves and particle beams is still absent. To this end, this review aims to bridge this gap by thoroughly exploring the characteristics of various ionizing radiation techniques and underscoring the recent progress of ionizing radiation-mediated synthesis and tailoring of inorganic nanomaterials for energy conversion. We begin with a concise overview of the principles behind radiation technology. Next, we discuss the controlled synthesis and modification of inorganic catalysts with various radiation sources, detailing radiation effects on the catalysts, including defect engineering, phase transformation, heterostructure formation, element doping, band gap engineering, morphology control, and so on. Following this, we highlight recent advances in irradiated catalysts for photo-/electrocatalytic applications, emphasizing the significant contributions of irradiation technology in energy and environmental protection. Finally, we articulate our perspective on the prospects and challenges of using ionizing irradiation technology.

## 2 Fundamentals of radiation technology

### 2.1 Fundamental concepts

**Fig. 2:**
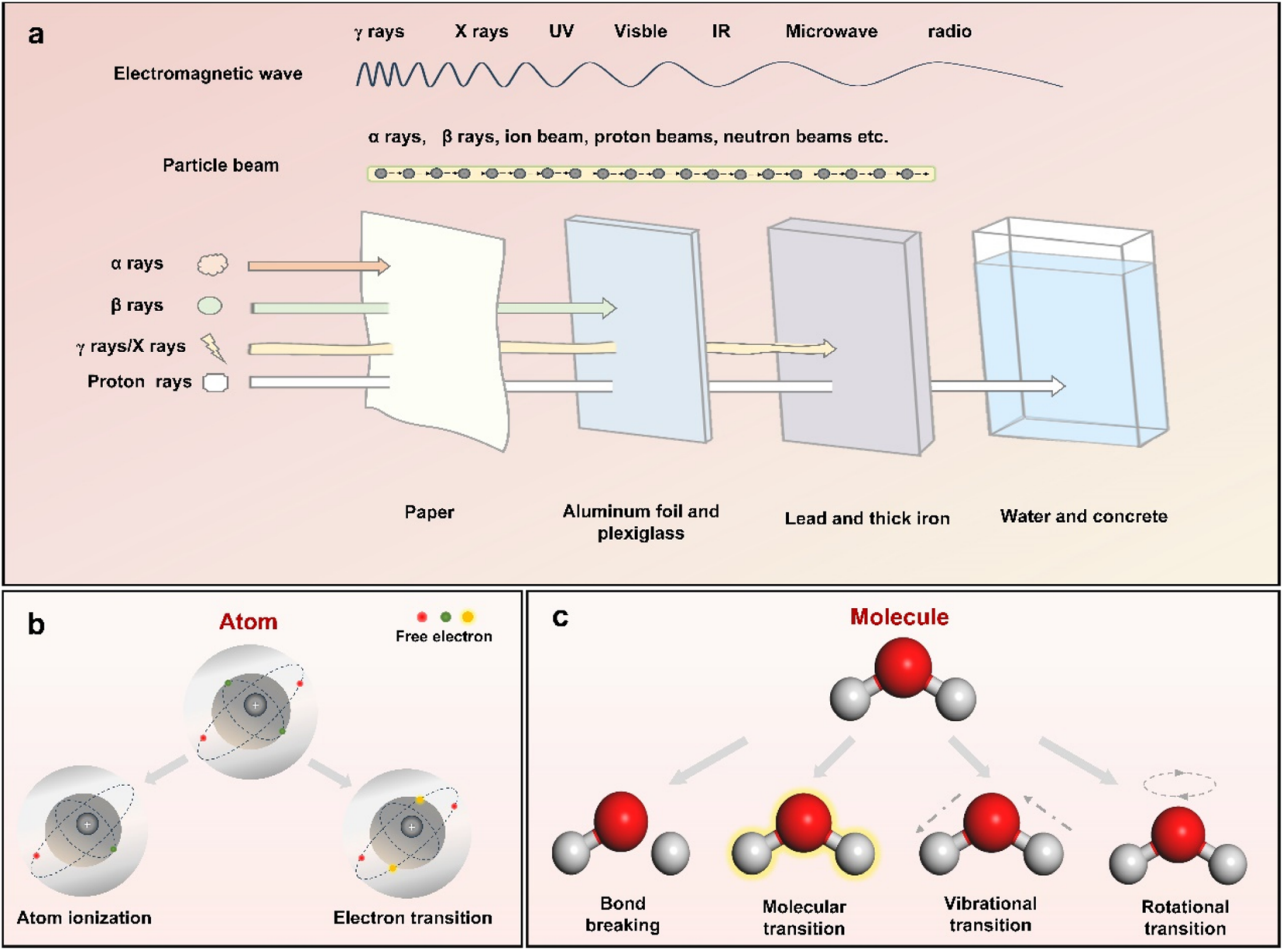
(a) Schematic illustration of the electromagnetic waves and particle beams accompanied by a comparison of their penetrating capabilities. An overview of the six effects of ionizing radiation on (b) atoms and (c) molecules.^16^

The term “radiation” refers to the transfer of energy through space or any other medium in the form of electromagnetic waves or subatomic particles (Fig. 2a). In the case of electromagnetic radiation, the spectrum extends from the high-energy gamma (*γ*) rays at one end to long-wave radio waves at the other, all of which propagate at the speed of light and are capable of transporting energy without requiring a physical medium. In contrast, particle beam radiation is made of atomic or subatomic particles, including protons, neutrons, electrons, and ions. These particles carry enough energy that can interact with atoms and molecules directly with different radiation effects, including radiolysis, photolysis, knock-on collisions and photothermal.^16,23^Fig. 2b and c show six common effects observed in substances exposed to different types of radiation sources. Atomic ionization, a key consequence of radiation, involves the ejection of electrons from atoms or molecules, typically caused by high-energy radiation, such as strong X-rays and alpha, beta and γ-rays. These radiation sources cause electrons in shell and nuclear orbitals to be released, as their photon energies exceed the binding energy of the electrons.^24–26^ On the other hand, lower-energy radiation, such as soft X-rays, causes only electron transitions to higher energy levels without inducing dislocation due to the absorption of photon energy by the core electrons.

The response of molecules to irradiation is determined by their structural characteristics. Typically, polar molecules can undergo ionization, leading to the formation of electron–hole pairs. In contrast, electronic excitation is more common in nonpolar molecules and leads to chemical bond dissociation. This phenomenon stems from the intrinsic interactions among electron orbitals that establish chemical bonds. As the energy of radiation extends into the UV, visible light, and infrared ranges, the photon energy becomes more closely aligned with the electronic and vibrational energy levels of molecules. This alignment facilitates molecular and vibrational transitions that promote photochemical reactions and the formation of reactive radicals. At lower energy levels, like those in the microwave range, the photothermal effect induces rotational transitions in polar molecules.

#### 2.1.1 Ionizing radiation

On the basis of energy levels, radiation can be divided into ionizing radiation and non-ionizing radiation. High-energy particle beams, made up of subatomic particles and ions and electromagnetic waves of γ-rays, X-rays, and high-frequency UV rays, are examples of ionizing radiation, all of which have the capacity to ionize electrons in atoms and molecules by ejecting electrons.^27–29^Table 1 presents a comparative classification of common irradiation techniques. The controlled adjustment of dose, fluence, and radiation source enables the synthesis and modification of nanomaterials with unique advantages for catalytic reactions. For example, ionizing radiation facilitates precise control over the size, shape, and crystallinity of the nanoparticles (NPs) without the need for hazardous chemicals, thus reducing environmental risks and costs. Additionally, it enables surface modifications, making the process more sustainable and energy-efficient.^19,21^ Additionally, ionizing radiation can intentionally introduce defects into catalysts. By adjusting parameters such as the intensity and energy of irradiating ions and irradiation time, the depth and concentration of defects within the catalysts can be controlled, which is a significant advantage over chemical methods to produce defects. These defects can modulate the electronic structure of the catalysts or generate new active sites, which is conducive to improving the catalytic performance. Finally, ionizing radiation is able to treat catalysts uniformly, ensuring consistency and reliability for scaling up to industrial production, thus expanding its practical application.

**Table 1:** Comparison and classification of common irradiation techniques

Category	Subcategory	Energy^a^	Source	Classification^b^
γ-Ray irradiation	−(<0.001 nm)	>1.24 × 10^6^ eV	Electromagnetic wave	Ionizing radiation
X-ray irradiation	Hard X-rays (0.001–0.1 nm)	1.24 × 10^4^–1.24 × 10^6^ eV	Electromagnetic wave	Ionizing radiation
	Soft X-rays (0.1–10 nm)	124–1.24 × 10^4^ eV	Electromagnetic wave	Ionizing radiation
Electron beam irradiation	Low energy	0.1–0.3 MeV	Particle beam	Ionizing radiation
	Medium energy	0.3–5 MeV	Particle beam	Ionizing radiation
	High energy	5–10 MeV	Particle beam	Ionizing radiation
Ion beam irradiation	Low energy ion irradiation	Less than 1 keV	Particle beam	Ionizing radiation
	Medium energy ion-irradiation	A few hundred keV to a few MeV	Particle beam	Ionizing radiation
	Swift heavy ion irradiation (SHII)	More than 1 MeV	Particle beam	Ionizing radiation
Neutron beam irradiation	Thermal neutron irradiation	∼0.025 eV	Particle beam	Ionizing radiation
	Cold neutron irradiation	5 × 10^−5^–0.025 eV	Particle beam	Ionizing radiation
	Fast neutron irradiation	0.1 MeV	Particle beam	Ionizing radiation
	Fusion neutron irradiation	14.1 MeV	Particle beam	Ionizing radiation
Proton beam irradiation	—	A few keV to a few hundred keV	Particle beam	Ionizing radiation
UV (ultraviolet)	Extreme UV (10–121 nm)	10.2–124 eV	Electromagnetic wave	Non-ionizing irradiation
	Far UV (122–200 nm)	6.2–10.2 eV	Electromagnetic wave	Non-ionizing irradiation
	Middle UV (200–300 nm)	4.1–6.2 eV	Electromagnetic wave	Non-ionizing irradiation
	Near UV (300–400 nm)	3.1–4.1 eV	Electromagnetic wave	Non-ionizing irradiation
Visible light	Visible light (400–700 nm)	1.8–3.1 eV	Electromagnetic wave	Non-ionizing irradiation
IR (infrared)	Near IR (0.7–1.4 μm)	0.89–1.8 eV	Electromagnetic wave	Non-ionizing irradiation
	Middle IR (1.4–3 μm)	0.41–0.89 eV	Electromagnetic wave	Non-ionizing irradiation
	Far IR (3 μm −1 mm)	1.2 × 10^−3^–0.41 eV	Electromagnetic wave	Non-ionizing irradiation
Microwave	-(1 mm -1m)	1.2 × 10^−6^–1.2 × 10^−3^ eV	Electromagnetic wave	Non-ionizing irradiation

a These energy ranges are typical and can vary depending on the specific application or source.

b There is no clear dividing line between ionizing and non-ionizing radiation in the spectrum of electromagnetic waves.

#### 2.1.2 Non-ionizing radiation

Compared to ionizing radiation, non-ionizing radiation, such as low-frequency UV rays, visible light, infrared light and microwaves, has lower photon energy. This type of radiation can excite electrons or molecules to higher energy states, facilitating molecular transition and rotation.^30^ Additionally, molecular vibrations and rotations are often accompanied by the release and absorption of thermal energy. For example, UV treatments are gentler and can effectively dissociate ligand bonds, which is favourable for promoting catalytic activity. The power, duration and wavelength of UV irradiation have a critical influence on the synthesis and modification of nanocatalysts.^30^ The irradiation of visible light is cost-effective, environmentally friendly, and safe, and it has been studied for decades. In contrast to UV and visible light, infrared photons lack sufficient energy to generate free radicals or electrons. Therefore, the synthesis and modification of catalysts by infrared radiation is primarily limited to infrared lasers. Although microwaves have higher photon energies, their primary role in catalyst synthesis is through thermal heating effects. Microwave radiation offers high efficiency and energy conversion rates but faces limitations in scalability. Based on these merits, non-ionizing radiation sources are more prevalent, less costly, and less hazardous than ionizing radiation, making them more common in laboratory settings.

### 2.2 Characteristics of different irradiation technologies

#### 2.2.1 γ-Ray and X-ray irradiation

Both γ-rays and X-rays are essentially composed of high-energy photons and have good penetration capabilities. However, their origins are different: γ-rays are emitted by the radioactive decay of atomic nuclei, while X-rays are produced when high-energy electrons or ions interact with a substance.^31^ In recent years, irradiation with both γ-rays and X-rays has been used for the synthesis of metal and metal compound NPs as well as carbon nanomaterials. This is because the γ-ray and X-ray radiation can induce radiolysis of the metal precursor solution, producing active substances such as electrons and free radicals that reduce or oxidize the metal ions to the corresponding elemental state or metal oxides. In addition, their high penetration capacity ensures uniform exposure and treatment of the entire precursor solution, thus promoting the nucleation and formation of NPs with different mass ratios. In this way, it can achieve the precise adjustment of particle size and distribution, which is critical for optimizing their physicochemical properties for a variety of applications.

**Fig. 3:**
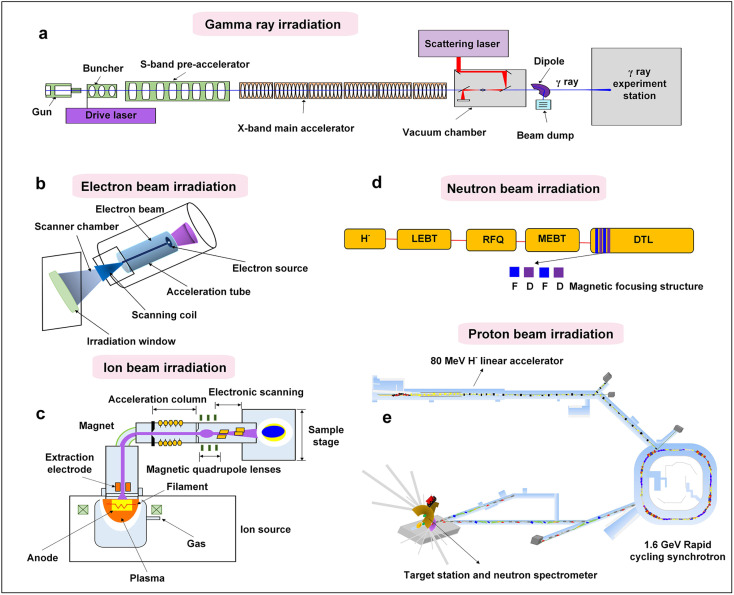
The principles and configurations of various types of irradiations: (a) overview of the construction of a generic electron beam accelerator; (b) structural design for a typical ion implanter; (c) configuration of advanced scattered neutron source accelerators; (d) structure of a proton accelerator; (e) general composition of a γ-ray source accelerator.

The overall configuration of γ-ray radiation is depicted in Fig. 3a, primarily including the components of the S-band photocathode microwave electron gun, convergence cavity, pre-acceleration section, and X-band main acceleration section. The γ-ray irradiation process begins with the accelerator generating a continuous beam, which is matched and focused. Laser pulses are generated by collision and scattering in the light source, resulting in the generation of a γ-ray pulse in the direction of the electron beam motion. Following the separation of the deflection magnet and electron beam, the generated γ-ray enters the γ-ray transmission section, where it is irradiated onto the substance through the collimation hole. Due to their high energy and strong penetration capabilities, γ-rays can pass entirely through the samples, providing uniform irradiation. The γ-ray irradiation process is straightforward to operate and can be conducted in atmospheric environments. Additionally, the sample station exposed to γ-rays is extensive, covering an area of several square meters.^32^ These characteristics position γ-ray irradiation as a promising way of realizing the development of highly efficient, cost-effective, mass-produced, and chemically stable catalysts.

#### 2.2.2 Electron beam irradiation

Electron irradiation accelerators are pivotal tools in radiation processing, primarily using high-energy electrons to treat various materials. As shown in Fig. 3b, a typical electrostatic accelerator includes key components such as an electron source, an acceleration tube, and an irradiation window. Operating in a vacuum, the accelerator propels the emitted electron beam, or X-rays, through the tube, accelerating them to high speeds. The irradiation window and the scanning system are then responsible for scanning the accelerated electrons/X-rays to the required irradiation width, ensuring that the irradiation energy is precisely delivered to the target material. A significant advantage of electron accelerators over other irradiation sources is that radiation dissipates immediately after shutdown, eliminating the risk of environmental contamination. In addition, the dose rate of the electron accelerator source is significantly higher than that of the ^60^Co source, reaching 3 to 4 orders of magnitude.^33–35^ It is clear that the high efficiency of electron accelerators greatly reduces the irradiation time and increases the yields, making them a key enabler for expanding the production process. Therefore, electron beam irradiation has the potential to play a crucial role in promoting the large-scale production of photo-/electrocatalysts.

#### 2.2.3 Ion beam irradiation

Ion beam irradiation, including ion implantation and ion irradiation, is an effective technique for modification of catalysts. Ion beam modification is typically categorized by energy level, including low-energy ion implantation (below 1 keV), medium-energy ion implantation (from tens of keV to several MeV), and high energy ion irradiation, such as swift heavy ion irradiation (above 1 MeV per amu). Fig. 3c illustrates the construction of a typical ion implanter, highlighting its four key components: the ion source, magnetic selection system, acceleration system and sample stage. In general, the basic process proceeds as follows: firstly, the hot electrons emitted from the ion source filament collide with gas molecules that are exposed to an electric field, resulting in ionization. The ions are then sorted using a magnetic analyzer based on their mass-to-charge ratio. The selected ions are accelerated through a tube to reach higher energy levels. Finally, the sample is exposed to the ion beam using a deflection scanner. The ion irradiation technique can be used to effectively tailor various catalysts by controlling the ion species, energies, fluence and irradiation angle.^36,37^ This technique offers distinct advantages, including high precision, favourable controllability, and reliable repeatability. Moreover, it allows for fine defect control and flexible element doping in numerous catalyst materials.

#### 2.2.4 Neutron beam irradiation

Neutron irradiation has distinctive characteristics, such as neutral charge, low magnetic moment, strong penetration capability, and a non-destructive effect on target samples. These properties make neutron beam irradiation an ideal tool for studying the static microstructure and dynamic behaviors of matter.^38,39^ In particular, significant progress has been made in spallation neutron sources driven by high-intensity proton accelerators, which have now entered the stage of practical application. As shown in Fig. 3d, spallation neutron source facilities consist of key elements such as a high-energy proton accelerator, a neutron target station, a neutron spectrometer, and the requisite supporting facilities. The principle behind this involves utilizing a high-energy, high-intensity proton accelerator to generate a high-power proton beam. The accelerator bombards heavy metals like tungsten (W) or mercury (Hg) with high-energy protons, triggering complex nuclear reactions, including intranuclear and extranuclear cascades, which produce a high flux of neutrons. Subsequently complex nuclear reactions, including intranuclear and exonuclear cascades of atoms, result in the production of high-fluence neutrons. Then, these neutrons are moderated and utilized for neutron irradiation research. Given its unique advantages, neutron irradiation has the potential to play an irreplaceable role in materials science, sustainable energy development, and other research domains.

#### 2.2.5 Proton beam irradiation

Proton irradiation distinguishes itself as a precise and controllable method for engineering nanomaterials. Its key advantage lies in the proton beam's ability to penetrate deeply with a well-defined range, determined by the Bragg peak phenomenon, where maximum energy deposition occurs. This property facilitates the creation of defects or the introduction of heteroatoms that can regulate band gap, enhance carrier separation, or increase the availability of active sites.^40^ As shown in Fig. 3e, proton irradiation technology commonly utilizes accelerators that include a front-end accelerator and a drift tube linear accelerator (DTL) main accelerator. The front-end accelerators consist mainly of an H^−^ ion source, low energy beam transport line (LEBT), radio frequency quadrupole accelerator (RFQ) and medium energy beam transport line (MEBT). The H^−^ ion source is accelerated by the RFQ to the MEBT, and finally to the DTL main accelerator. Notably, proton beam irradiation ensures a uniform dose distribution, making it particularly advantageous for large areas and irregularly shaped samples. Herein, high-energy protons enable the required modifications on the surface of catalysts, effectively adjusting the electronic and surface properties of catalysts with better catalytic performance.^41^

#### 2.2.6 UV irradiation/visible light/IR/microwave irradiation

Based on ionizing radiation discussed in the previous sections, including electromagnetic waves and high-energy particles that penetrate deep into catalysts and induce important atomic interactions, non-ionizing radiation, including ultraviolet (UV), visible, infrared (IR), and microwave irradiation, offers distinct advantages. These low-energy radiation features vary in photon energies and wavelengths, enabling controlled interactions with catalysts through excitation, vibration, and thermal mechanisms, rather than through direct ionization or high-energy particle impacts. Non-ionizing radiation methods are particularly valued for their accuracy, efficiency and adaptability in the synthesis and modification of nanomaterials, making them complementary tools in the design and enhancement of photo-/electrocatalysts.

Typically, UV wavelengths range from 10 to 400 nm, corresponding to photon energies between 3 and 120 eV.^16^ UV light can be further categorized into near-ultraviolet, mid-ultraviolet, far-ultraviolet and extreme ultraviolet, in descending order of photon energy. The power, duration, and wavelength of UV irradiation significantly impact the growth of NPs. For example, far-ultraviolet and extreme ultraviolet light can act as ionizing radiation, as its photon energy exceeds atomic ionization thresholds. In contrast, visible light, which spans a wavelength range of 400–700 nm, interacts with catalysts in a different manner. Typically, visible light has photon energies between 1.8 and 3.1 eV, which is perceptible to the human eye.^42^ Similar to UV irradiation, visible light can ionize solvents through multiphoton effects, generating reactive groups and solvated electrons. Visible light irradiation is cost-effective, highly controllable, environmentally friendly, and safe, and researchers have been experimenting with this technique for decades. Moving further down the spectrum, infrared (IR) light features even lower photon energies and distinct interaction pathways. Infrared light has a lower photon energy than UV light, with wavelengths ranging from 700 nm to 1 mm, and is further classified into near-infrared, mid-infrared, and far-infrared. Infrared frequencies align closely with the vibrational and rotational frequencies of molecular bonds, allowing infrared photons to be absorbed or emitted by molecules during vibrational or rotational transitions. Different from UV and visible light, infrared photons lack sufficient energy to generate free radicals or electrons, thus limiting their use for the synthesis and modification of inorganic nanocrystals. At the longest wavelengths of the non-ionizing spectrum, microwaves offer unique thermal capabilities. Microwaves, with wavelengths between 1 mm and 1 m and photon energies from 1.24 MeV to 1.24 μeV, differ significantly from higher-energy portions of the electromagnetic spectrum. Microwaves are primarily used for the thermal heating of nanomaterials due to their high efficiency and energy conversion rates, though scalability remains a limitation. Non-ionizing irradiation plays an integral role in the synthesis of metals, metal compounds, and carbon materials.^43–45^ One of the common advantages shared by both non-ionizing and ionizing irradiation is their high versatility across various materials, eliminating the need for conventional heating equipment.^16^ Consequently, the issues associated with inhomogeneous heat transfer, commonly encountered in traditional wet chemistry, can be effortlessly alleviated.

### 2.3 Important factors for the radiation process

#### 2.3.1 Irradiation energy (*E*)

Irradiation energy, referred to as E, determines the depth to which the implanted particles penetrate the material matrix. Typically, this energy ranges from a few kiloelectron volts (keV) to several megaelectron volts (MeV), resulting in implantation depths from a few tens of nanometers to a few micrometers. Moreover, the penetration depth implanted into a material is linearly proportional to the incident energy.

#### 2.3.2 Irradiation fluence (*j*)

The irradiation fluence is usually represented by *j* and is defined as the number of particles introduced into the surface unit of the material per unit time. It can be expressed by formula (1):

1 *j* = *N*/*t*

where *j* is the irradiation fluence, *N* represents the number of implanted particles, and *t* is the irradiation time.

#### 2.3.3 Radiolysis and G-values

Radiolysis is a radiochemical change that describes the dissociation of molecules in the presence of radiation. The process of radiolysis of substances is very complex. Radiolysis products can be a combination of the following: subatomic particles, free radicals, ions and molecules, and to quantify these radiolysis products, the concept of the *G*-value is employed, which represents the number of molecules, atoms, or radicals produced (or consumed) per 100 eV of absorbed energy. Among all substances, water is the most extensively studied in terms of radiolysis, with established *G*-values provided in parentheses, as shown in formula (2).2
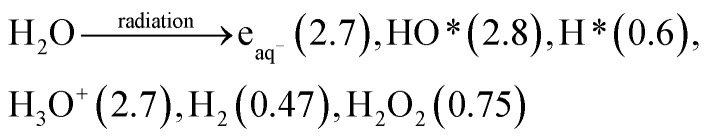
When water absorbs ionizing radiation, it produces cations, excited molecules, and electrons, which are distributed along the path of the incident particle, creating a localized high-energy region. These primary products undergo rapid transformations, including excited molecule dissociation, ion–molecule reactions, and electron solvation processes to form hydrogen atoms, hydroxyl radicals, and hydrated electrons. Some free radicals interact to form stable molecular products, such as hydrogen and hydrogen peroxide, or recombine to regenerate water molecules. The remaining unreacted radicals diffuse outward as the spike expands, eventually achieving uniform distribution in the aqueous medium.^16^ The extent of radiolysis depends on several factors, including the type and energy of the radiation, dose rate, impurities in the system, scavengers, irradiation environment (such as temperature and atmosphere), and the nature and state of the irradiated material.

#### 2.3.4 Absorbed dose (Gy) and dose rate

The absorbed dose is defined as the amount of energy imparted by ionizing radiation per unit mass of a material. This dose is crucial in understanding the response of the catalyst to irradiation and is commonly measured in units of grays (Gy) or kilorads (krad), where 1 krad is equivalent to 10 Gy. The absorbed dose quantifies the ionizing energy that is transferred to the catalyst, which can cause molecular changes, including ionization and excitation events within the irradiated substance. In addition to the absorbed dose, the dose rate describes the rate of energy delivery over time, typically expressed in units of Gy s^−1^. The dose rate is particularly important in radiolysis studies, as it affects the distribution and stability of radiolytic species, and thereby the efficiency of synthesis or modification processes. Together, the absorbed dose and dose rate serve as critical kinetic parameters that determine the yield, concentration, and overall quantity of active species produced. These active species play pivotal roles in chemical reactions, facilitating the precise synthesis and functional modification of catalysts.

## 3 Synthesis and tailoring of inorganic nanomaterials with ionizing radiation

### 3.1 Material synthesis

The synthesis of inorganic nanomaterials through radiation represents a cutting-edge way to modify chemical compositions and catalyze atomic-level reactions in solution by using high-energy particles or photons. By carefully controlling irradiation parameters, such as energy, fluence and ion species, the size, morphology and crystallinity can be well controlled, enabling the tailored preparation of catalysts beyond the reach of traditional methods. This process entails irradiating a precursor solution in which solvated electrons and free radicals generated by radiation drive reduction or oxidation reactions, culminating in the nucleation and growth of NPs. Therefore, ionizing radiation synthesis offers distinct advantages, including uniform size distribution, minimized aggregation, and improved purity. In addition, it can operate under ambient conditions and avoid the use of toxic chemicals, making it an efficient and environmentally friendly approach that is essential for the sustainable synthesis of nanocatalysts.

#### 3.1.1 γ-Ray and X-ray irradiation

Despite the risk associated with radiation, researchers have shown strong interest in using γ-rays and X-rays for nanomaterials synthesis due to their unique benefits. The primary advantages of γ-ray and X-ray irradiation are simplicity, low cost and zero emission. Typically, the process involves placing a prepared solution in an irradiation chamber, setting irradiation parameters, and allowing the sample to undergo irradiation. Compared to X-rays, γ-rays have higher energy levels, enabling a wider range of applications in nanomaterials synthesis.

In a pioneering study conducted in 1962, Yamazaki and colleagues investigated the effects of γ-rays on the synthesis of Au nanoparticles. They observed the formation of Au sol from an aqueous solution of chloroauric acid under γ-ray irradiation with ^60^Co as the radiation source.^46^ Building on this foundational work, subsequent studies have successfully synthesized both mono- and bimetallic nanoparticles using a similar strategy,^47–49^ which has led to widespread exploration of γ-ray irradiation for the synthesis of metal-based nanomaterials. For instance, Kianfar *et al.* synthesized Pt NPs/reduced graphene oxide (rGO) hybrids using γ-radiation. The rGO served as an electron sink, facilitating the formation of Pt nanoclusters and increasing the number of active sites.^50^ They observed that the particle size ranged from 1–8 nm, with an average size of 4.11 nm at an acid ratio (sulfuric to phosphoric acid) of 100. However, at a lower acid ratio of 50, the average size of particles increased to about 71.09 nm. This results indicated that a combination of reducing agents and irradiation creates a highly reducing environment, which favors metal precursor reduction and promotes seed formation and NP growth.^16,51^ Extending the versatility of γ-ray irradiation synthesis, Yu and colleagues prepared Vulcan xc72-supported Pt NPs by γ-radiation, NaBH_4_ reduction and polyol reduction methods, respectively.^52^ They found that the Pt NPs prepared by γ-radiation exhibited higher ORR activity and Pt utilization, even better than that of commercial Pt/C catalysts. Wang *et al.*^53^ also reported the preparation of Pt-decorated MoS_*x*_ catalysts using a two-step γ-ray radiation-induced reduction method in an ethylene glycol medium. The γ-radiation synthesis approach offered a shorter reaction time and higher yield, demonstrating its potential for practical applications.

Most recently, electron beam irradiation has also been utilized for the synthesis of metal–organic frameworks (MOFs). For example, Zhang *et al.*^56^ reported the synthesis of HKUST-1@Cu_2_O heterostructures using γ-radiation under ambient conditions, which exhibited superior catalytic activity in the reduction of *p*-nitrophenol to *p*-aminophenol to the pristine HKUST-1. This approach offers advantages in terms of convenience, environmental compatibility, and significant time and energy savings. Furthermore, the γ-radiation-induced post-synthesis technique can be applied to other crystalline porous materials to produce a variety of derivatives, thus expanding its applicability across diverse fields.

Despite the numerous advantages of γ-ray and X-ray irradiation in the synthesis of inorganic nanomaterials, gaining a deeper understanding of the mechanisms underlying crystal formation is essential, as it’s crucial for further optimizing their properties and enabling precise control over the synthesis process to achieve desired performance. A key factor in achieving efficient synthesis through γ-ray and X-ray irradiation lies in the modulation of radiation parameters. Among different parameters, precursor concentration plays a significant role, because it influences the final shape, size, and dispersion of NPs. Theoretically, higher concentrations of precursor tend to produce larger particles with lower dispersion, as the nuclei have more reactive atoms available in solution, promoting growth of nanocrystals. However, the effects of irradiation dose on nanoparticle size remain a subject of ongoing research. Some studies reported a decrease in particle size with increasing dose.^57,58^ For example, Wiguna *et al.*^59^ synthesized Ag NPs using γ-ray irradiation. TEM images showed that the Ag NPs were spherical in shape and the average size became larger with increasing irradiation dose. However, others found that particle size increased with the decrease of dose. This variation highlights the need for precise dose control to optimize particle characteristics. In addition to dose, the dose rate is another critical parameter that influences the growth habits of NPs. The dose rate can affect metal atom production rate, thereby influencing the size and shape of resulting products. According to classical nucleation and growth theory, a higher dose rate leads to a faster production of metal ions, increasing atomic concentration and supersaturation, which reduces critical nucleus size.^60^ Consequently, smaller sized nuclei form at higher dose rates, resulting in smaller sized nuclei. Recent studies further emphasize the importance of dose rate control in tailoring nanoparticle characteristics. For example, Kepić *et al.* used low γ-ray doses (1–20 kGy) to prepare Au NPs anchored on RGO sheets, and they observed that higher doses promoted larger particle growth (at 5 and 10 kGy) or a broader particle size distribution (at 20 kGy).^61^ This result demonstrates that the control of dose rate is important to achieve the desired size and distribution during the process of irradiation-based synthesis.

#### 3.1.2 Electron beam irradiation (e-beam radiation)

Electron beam irradiation has been demonstrated to be a versatile and potent technique for the synthesis of nanomaterials. Using high-energy electron beams induces structural changes and catalyzes chemical reactions, providing precise control over the formation of nanomaterials. Through careful modulation of the electron beam parameters, the sizes, shapes, and crystallinity of NPs can be well controlled. A distinct advantage of electron beam irradiation lies in its ability to deliver energy directly to targeted regions. This localized energy delivery enables the synthesis of nanomaterials without the need for high-temperature conditions or harsh chemical environments, making the process both efficient and mild. Thus, electron beam irradiation can enhance the uniformity and purity of catalysts, while significantly reducing the cost. Moreover, e-beam radiation aligns with the principles of green chemistry by reducing energy consumption and minimizing waste production.

Recent empirical studies underscore the efficacy of e-beam irradiation for the synthesis of nanomaterials. For example, Lee and colleagues successfully prepared PVP-stabilized Cu NPs in CuSO_4_ solution dissolved in isopropanol (IPA) by e-beam radiation.^62^ By controlling the beam energy, beam current, and absorbed dose, the particle sizes of the Cu NPs were well tuned. More specifically, they observed that higher beam energy and beam current produced smaller particles, while an increase in radiation dose led to larger particle sizes. Similarly, Zhou *et al.* prepared colloidal Ag NPs from an aqueous AgNO_3_ solution using e-beam irradiation. Their results demonstrated that a higher dose rate was necessary to achieve optimal yields of Ag NPs, which underscores the importance of dose rate control in maximizing nanoparticle production. To further explore the comparative efficacy of electron beam and γ-ray irradiation in radiosynthesis, Nguyen *et al.* synthesized gold nanoparticles (Au NPs) by exposing an aqueous HAuCl_4_ solution to both e-beam and γ-ray radiation.^63^ The results from this work showed that the Au NPs synthesized with e-beam irradiation were significantly smaller than those produced using γ-rays at an equivalent dose rate, highlighting the enhanced precision of e-beam irradiation for achieving finer particle sizes.

**Fig. 4:**
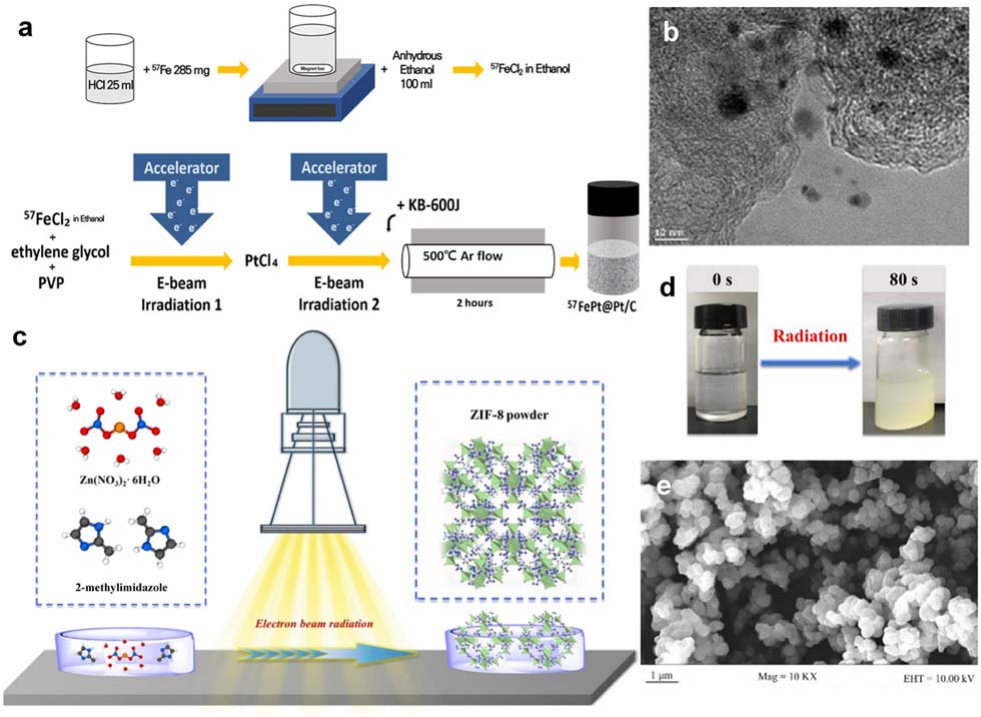
(a) Schematic illustration of the synthesis of 57FePt@Pt/C *via* electron-beam irradiation. (b) TEM image of 57FePt@Pt/C. Copyright 2023 Wiley-VCH. (c) The synthesis setup of ZIF-8 *via* electron beam radiation. (d) The photo of ZIF-8 synthesized at an absorbed dose of 50 kGy. (e) SEM image of ZIF-8 at an absorbed dose of 50 kGy.^54,55^ Copyright 2022, Wiley-VCH.

Electron beam irradiation has also been demonstrated to be effective in constructing complex core–shell structured nanocatalysts. For instance, Lee *et al.* used a two-step e-beam irradiation process to fabricate 57FePt@Pt core–shell structures, followed by heat treatment to produce 57FePt@Pt/C catalysts.^54^ As depicted in Fig. 4a, the Fe core is initially formed by a first e-beam irradiation at 80 kGy, followed by a second e-beam irradiation at 40 kGy to form a Pt shell layer. The TEM image of Fig. 4b confirms the successful synthesis of the core–shell structure. The rapid formation of the 57FePt@Pt/C core–shell structure and its excellent electrocatalytic activity demonstrate the suitability of e-beam irradiation for synthesis.

Beyond inorganic nanomaterials, e-beam irradiation has also shown promise in the synthesis of MOFs. Most recently, Chen *et al.*^55^ synthesized ZIF-8 by using high energy e-beam irradiation (Fig. 4c–e). The cumulative absorbed dose was 50 kGy in 80 s. For comparison, ZIF-8 can also be synthesized using conventional solvent heating at 140 °C for 24 h to achieve similar crystallinity and yield. Notably, the energy consumption for e-beam irradiation was two orders of magnitude lower than that required under solvent-heated conditions, suggesting that e-beam irradiation offers ultra-rapid synthesis with significant energy savings. This is because the e-beam radiation effectively activates the whole reaction system, forming highly reactive anionic radicals, leading to a reduction of the energy barrier and significantly increasing the nucleation rate of ZIF-8 nanocrystals. The versatility of e-beam irradiation is further demonstrated in the synthesis of other MOFs, such as MIL-53, MIL-101, and MOF-73, under conditions similar to those used for ZIF-8, proving the effectiveness and broad applicability of e-beam irradiation for the synthesis of MOF materials. In addition, e-beam irradiation has shown potential for synthesizing covalent organic frameworks (COFs). For instance, Wang's group demonstrated that COFs can be rapidly synthesized *via* e-beam irradiation, with various products obtainable within minutes under ambient conditions.^64^ This strategy requires only a single radiation source and minimal equipment, presenting a scalable and high-throughput strategy that holds promise for revolutionizing the industrial production of MOFs and COFs.

#### 3.1.3 Non-ionizing irradiation

Similar to ionizing radiation, non-ionizing radiation is versatile for synthesizing a wide range of nanomaterials.^16^ However, non-ionizing radiation primarily interacts with substances at the molecular level. In all cases, if not reflected, photon energy is absorbed, exciting molecular motion and heating the substance. Consequently, non-ionizing radiation can produce both photothermal and photochemical effects. When matter is heated to extreme temperatures, plasma may form, with electrons in the plasma potentially inducing further chemical reactions. Due to these effects, it is often challenging to elucidate the precise mechanisms involved during the synthesis and modification process. Moreover, UV, infrared, and visible light irradiation often require photoinitiators, while microwave radiation generates thermal shock waves. The transient rise and fall of high temperatures make the control of size and shape challenging, often resulting in a broader size distribution. Despite these challenges, non-ionizing radiation remains a promising tool for the efficient synthesis of nanomaterials. With growing interest, extensive research is being conducted in this area, indicating substantial potential and room for further development.

### 3.2 Material modifications

#### 3.2.1 Defect engineering

The presence of defects in catalysts exerts a substantial influence on their catalytic activity. Most recently, numerous research works have demonstrated that defect engineering not only activates the host substrate but also generates more sites, which are beneficial for photo-/electrocatalysis reactions.^65,66^ Among various methods, ionizing irradiation stands out for being an impurity-free, high-energy and momentum-driven approach. These distinctive merits render it a promising way to introduce defects into catalysts. Specifically, when energetic particles pass through solid matter, they collide with the nuclei and electrons of the catalyst, transferring kinetic energy. If the recoil atoms in the target gain enough kinetic energy to deviate from their original positions, various atomic-scale defects such as point vacancies are created. Although most point defects disappear in sub-microsecond time, some defects persist in the system. Additionally, the electronic structure of the material strongly influences the outcome of electron or ion collisions due to the varying mechanisms by which electronic excitations are converted to heat. In materials with conducting electrons, the action of electronic excitations in nanosystems differs from that in bulk materials due to the different mechanisms. In materials with conducting electrons, the electronic excitations are dispersed, thus reducing their impact. Thus, radiation damage is mainly caused by atomic displacements. In contrast, in insulators, excitation can lead to localized bond breaking. These varied responses of these systems to radiation can be exploited to selectively modify the structure of composites, allowing the tailoring of catalysts with excellent electrochemical properties.

The interaction of irradiation with nanoscale systems often leads to the formation of defects, which can impose size constraints in one or more dimensions. These resulting defects can either enhance or restrict their catalytic behaviours, depending on their nature and distribution. Particularly, in some small-volume nanosystems, electronic excitations generated by irradiation, such as those caused by high-energy electrons or particles, tend to disperse more rapidly. This dispersion reduces their localized impacts, thereby diminishing the overall effects of irradiation in such systems. Therefore, in metallic nanosystems, radiation damage is predominantly caused by atomic displacements, leading to the formation of vacancies, interstitials, or other structural defects. In contrast, in insulators, excitation can lead to localized bond breaking. The different responses of these systems to radiation can be exploited to selectively modify the structure of composites, allowing the tailoring of catalysts with excellent photoelectrochemical properties. However, in bulk systems, all energy is eventually absorbed.^67^ Thus, when energies are high, nanoscale damage is relatively minor as long as displacement cascades do not play a significant role. However, it is not the case that smaller material sizes are better. It is worth noting that the reduction in size leads to a different temperature distribution, resulting in localized temperatures above the melting point, which may have a significant impact on nano-systems, especially in processes such as nanoscale material modification and fabrication. The selection of nanomaterials with appropriate sizes and further exploration of the behaviour of nanosystems under irradiation with energetic particles is crucial for optimizing and controlling the formation of defects, as well as for exploiting the unique properties and functionalities of nanomaterials for various catalytic applications.

For ion beam irradiation, when the incident ions sweep over the surface of the sample, a small number of atoms recoil from the surface. The heavier ions are used to hit the lighter nuclei from the sample and probe the recoil atoms so that the depth distribution of the recoil atoms in the surface region can be obtained. Meanwhile, the deceleration of high-energy ions as they pass through solid matter can be categorized into two mechanisms: electronic stopping and nuclear stopping. A nuclear stopping occurs when the ion collides with the nucleus of the target atom, transferring kinetic energy to the target atom and resulting in translational motion of the target atom. The energy loss is determined by screening the Coulomb interaction. It should be noted that nuclear stopping is only effective for relatively slower and heavier ions of all types. In contrast, electron stopping is influenced by inelastic collisions between moving ions and target electrons, which can be either bound or free. This type of stopping is the result of various physical processes, including ionization of target atoms, electron–phonon coupling, and collective electronic excitation such as plasma. At higher ion energies, electron stopping becomes dominant. The transition from nuclear to electronic stopping depends on the ion mass. For hydrogen ions (protons), electronic stopping is dominant.

**Fig. 5:**
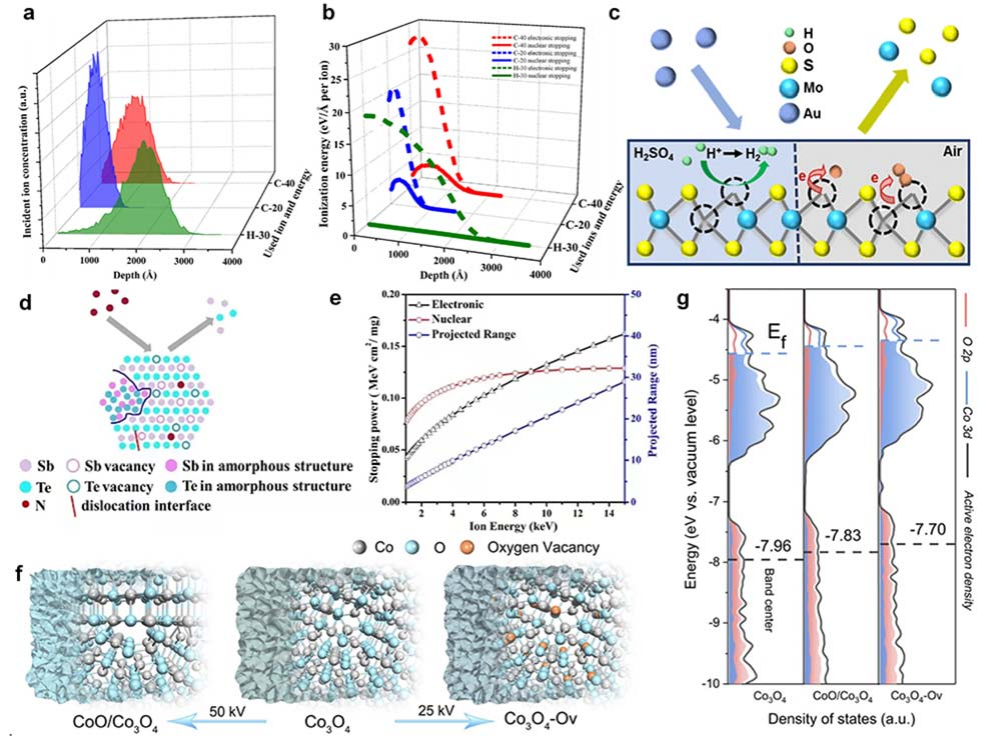
SRIM simulations of ion irradiation effects on MoS_2_ using H-30 keV, C-20 keV and C-40 keV. (a) The depth of ion density penetration for each ion type; (b) the analysis of electronic and nuclear stopping powers for the irradiated ions; Copyright 2013 Elsevier Ltd. (c) Schematic illustration of electron transfer from MoS_2_ to O_2_ adsorbed at defect sites. Copyright 2018, American Chemical Society. (d) Schematic illustration under N^+^ irradiation. (e) The relationship between nuclear stopping powers, electronic stopping powers and projected range under N^+^ irradiation. Copyright 2019, Elsevier Ltd. (f) Schematic illustrations of the formation of Co_3_O_4_, Co_3_O_4_-O_v_, and CoO/Co_3_O_4_ samples. (g) PDOS plots.^28,68–70^ Copyright 2020, Wiley-VCH.

Recently, Mravik *et al.* reported the irradiation of MoS_2_ nanopowder with C^2+^ ions (energy 20 keV and 40 keV and ion fluences of 5 ×10^14^ and 10^16^ ions per cm^2^) and H^+^ ions (energy 30 keV and ion fluences of 10^16^ and 10^17^ ions per cm^2^).^68^ Stopping and Range of Ions in Matter (SRIM) calculations were performed to understand the effects of different ion irradiation sources on the MoS_2_ structure. It was found that the lighter H^+^ ions penetrate deeper compared to the heavier C^2+^ ions (Fig. 5a). At 20 keV and 40 keV energies, H^+^ ions produce only 6 vacancies per ion, while C^2+^ ions produce 189 and 304 vacancies per ion, respectively (Fig. 5b). Typically, incident ions can be halted on the target material by either electronic or nuclear stopping. Thus, the effect of ion irradiation on the material depends on whether the stopping mode predominates. Electronic stopping leads to ionization and electronic excitation, while nuclear stopping causes bond breaking, atomic displacement, vacancy creation and phonon excitation. It can be seen that the main energy loss mechanism for H^+^ ions is ionization, and therefore H^+^ ions are predominantly blocked by electrons, while nuclear blocking is negligible. If the incident ion is a C^2+^ ion, the main energy loss mechanism is also ionization, but there is a significant amount of energy transfer to the target lattice (phonon excitation) and recoils and successive cascades of Mo and S. The main energy loss mechanism is the ionization of the carbon ion. Thus, electronic stagnation dominates, but the contribution of nuclear stagnation is also significant, which is consistent with higher vacancy production. When irradiating a material, the different ion penetration depths in the material may have different effects on the number of vacancies, where the intrinsic reaction mechanisms vary, while the effects of ion energy, fluence and incidence angle on defects such as vacancies remain to be explored.

Luxa *et al.*^69^ investigated the impacts of radiation (S, Se, Te) on the electrocatalytic performance of bulk MoS_2_ nanosheets. The study was conducted with ionic fluences ranging from 1 × 10^14^ to 1 × 10^16^ ions per cm^2^ at a mean ion energy of 400 keV. Exploring the penetration of ions in materials provides valuable insights into the formation of defects and the distribution of defect concentrations. Thus, SRIM simulations were carried out to investigate the ion beam penetration of MoS_2_. The simulation projected range (the depth at which the maximum ion injection occurs) decreases as the ion mass is increased. The range of individual projections varies as S (338 nm) > Se (162 nm) > Te (105 nm). He *et al.*^28^ fabricated a single layer of MoS_2_ using 500 keV high-energy Au-ion irradiation. They found that ion irradiation primarily generated S vacancies. As the defect increases, the photoluminescence (PL) peak position shifts from high to low energy levels due to the electron transfer from MoS_2_ to the adsorbed O_2_ at the defect sites, resulting in electron depletion and hole enrichment (Fig. 5c).

In addition, the modification of catalysts with ion irradiation is inextricably linked to different electron stopping/nuclear stopping mechanisms. Typically, electronic stopping leads to the ionization of the target by releasing electrons, while nuclear stopping leads to bond breaking, atomic displacement and vacancy formation. It is also important to consider the case of electron stop/nuclear stop crossover. Fig. 5d illustrates the interaction between electron stopping and nuclear stopping, as evidenced by SRIM simulations. The simulations show that the nuclear stop is about 10 times higher for Te ions compared to S ions, and that the nuclear stop is dominant for Te ions, whereas the electronic stop is significant for S and Se ions. In addition, vacancy depth profiles were simulated and displacement energies were calculated to estimate the formation of vacancies (Fig. 5e). The results show that ion irradiation mainly leads to preferential sputtering of S atoms, resulting in S vacancies. In addition, numerical simulations of the vacancy depth profiles show that S vacancies are predominantly produced in the case of Se and Te ion bombardment, whereas this effect is much smaller in the case of bombardment with S ions. Moreover, the formation rate of Mo vacancies is significantly lower due to the higher displacement energy value of Mo. Drawing from the aforementioned findings, it is evident that comprehending interactions between the electronic and nuclear stopping mechanisms and their effects on vacancy formation is critical for customizing ion irradiation processes to achieve specific modifications in material properties.

In Huang's study, N^+^ ion irradiation was used to modify the crystal and electronic structure of Sb_2_Te_3_ nanoplates.^71^ A series of characterization results revealed that the electronic structure changes induced by irradiation are closely linked to electron stopping and nuclear stopping processes. The relationship between electron stopping, nuclear stopping, incidence range, and energy of N^+^ ions was further evaluated with SRIM (2013) simulations. The simulation results indicated that the kinetic energy of N^+^ ions is dissipated through both electronic and nuclear stopping mechanisms, with a primary implantation depth of 29 nm. That's to say the N-doping enhanced the intrinsic activity of the Sb_2_Te_3_ catalyst by altering the electronic structure of the surface atoms. Additionally, the electrocatalytic activity and electronic structure of catalysts can be adjusted by introducing various types of defects and increasing the number of dangling bonds around the active site. In particular, the unique ion beam sputtering effect not only strips the surface of the catalyst, but also increases the degree of catalyst roughening, and this irradiated sputtering effect dramatically increases the specific electrochemical area of the catalyst, providing more active sites.

In a recent study, He *et al.*^70^ demonstrated the effective regulation of the surface-active electron density of Co_3_O_4_ through an argon-ion irradiation method. Through a combination of DFT calculations and characterization of the ultraviolet photoelectron spectrometer spectrum, a notable upshift in the band center of Co_3_O_4_ was unveiled (Fig. 5f and g). This shift substantially enhanced the adsorption capacity of the oxygen-containing groups and reduced the barrier for the OER, leading to enhanced performance toward water splitting. These results demonstrated that ion radiation could effectively optimize the surface electron density of catalysts, holding the potential for shaping the future rational design and discovery of superior devices.

Remarkably, ion irradiation has demonstrated remarkable efficacy in augmenting the volume of interfacial area through defect and interface engineering, which significantly enhanced their catalytic performance. Recently, Sun *et al.*^72^ tuned and optimized the defects and interfacial engineering on PtPb nanoplates by C^+^ ion irradiation. By adjusting the C^+^ ion fluence, the crystalline phase of PtPb nanoplates was transformed from single crystal to polycrystalline, while the surface layer of PtPb nanoplates was distributed with different degrees of dislocation and subcrystalline boundaries, and some of them were even amorphized. Electrochemical measurement results indicated that irradiation-treated PtPd nanoplates with defects and single-crystal/polycrystalline interfaces and appropriate amorphous phases are favourable for the ORR. In a related study, the same group investigated PtPb nanoparticles under nonaqueous conditions through 1 MeV Kr^3+^ ion irradiation.^73^ It was found that Kr^3+^ ion irradiation induces the formation of a crystalline/amorphous phase interface characterized by a unique electronic structure. This interface consisted of a crystalline phase surrounded annularly by an amorphous phase, playing a dominant role in lowering the reaction barrier. DFT calculations further confirmed that the novel interface activated the C–H and O–H bonds, optimized the adsorption of hydroxyl groups and intermediates on the surface and promoted the oxidation reaction, ultimately exhibiting enhanced electrocatalytic activity.

**Fig. 6:**
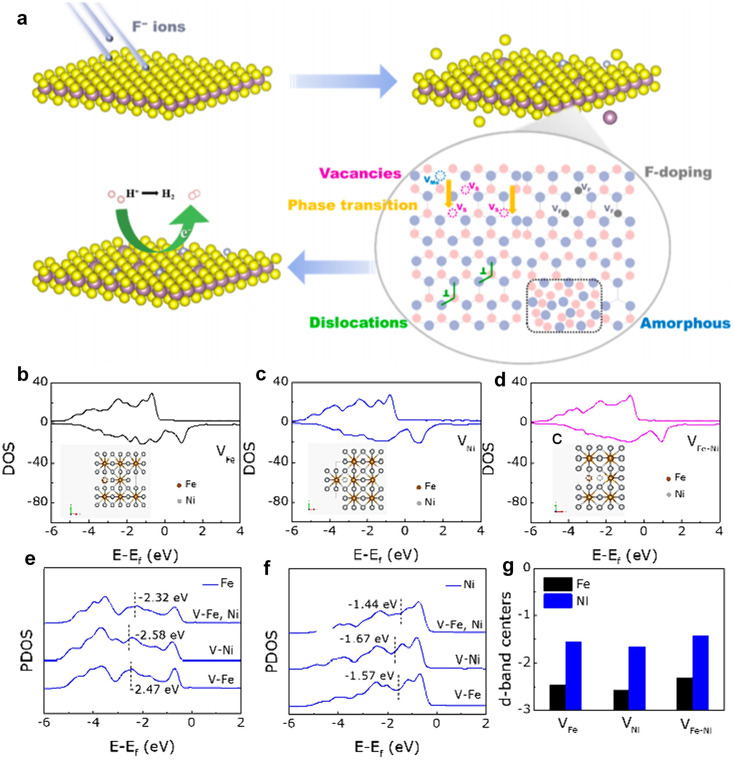
(a) Schematic illustration of the irradiation process of MoS_2_ structure evolution. Copyright 2023 American Chemical Society. DOS of (b) FeNi_3_–V_Fe_, (c) FeNi_3_–V_Ni_, and (d) FeNi_3_–V_Fe_–_Ni_. PDOS of (e) Fe and (f) Ni atoms under three different structures. (g) Changes in the d-band center. Copyright 2024 Elsevier Ltd.

Dong *et al.* used a controlled flux of F-ion beams to irradiate MoS_2_, combining doping and defect engineering to investigate whether F-ions can effectively modulate the electrical structure of MoS_2_ for better HER performance.^74^Fig. 6a illustrates the schematic structure evolution of MoS_2_ under irradiation. The pristine MoS_2_, with a small number of sulfur vacancies, exhibited poor HER catalytic activity. As the ion fluence increased, the concentration of F doping and sulfur vacancies increased proportionally, reaching optimal HER catalytic activity at a fluence of 2 × 10^13^ ions per cm^2^. However, when the fluence is further increased to 5 × 10^13^ ions per cm^2^, a large number of disordered regions and subgrain boundaries were generated, resulting in a suppression of HER performance. Additionally, the reduced crystallinity of MoS_2_ nanosheets led to an increased number of grain boundaries, with sulfur vacancy boundaries located within or near these grain boundaries being less catalytically active, thereby diminishing HER performance. Moreover, when the concentration of sulfur vacancies became too high, the intrinsic activity of the sites decreased. Although the number of vacancies correlated positively with ion fluence, excessive lattice symmetry disorder and reduced crystallinity significantly hindered catalytic performance.

Felix *et al.*^75^ provided the first comprehensive study on the effects of γ-radiation on the structural, optical, and magnetic properties of monolayer WS_2_ nanosheets, highlighting significant alterations induced by γ-ray exposure. It was found that the interactions in radiometry, such as photoelectric absorption, Compton scattering, pair production, vacancies and fast electrons, played an essential role in modifying these properties. Collectively, these processes lead to notable changes in the electrical, optical and structural properties of WS_2_ nanosheets. Experimental results confirmed that the γ-irradiation significantly affected the physical properties of WS_2_ nanosheets. X-ray photoelectron spectroscopy (XPS) results revealed that irradiation introduces defects, with defect density increasing alongside irradiation dose. Furthermore, Raman spectroscopy tests showed that as the γ-irradiation dose increased, a blue Raman shift of the A_1g_(Γ) peak was observed, along with an increase in the Raman intensity ratio between the A_1g_(Γ) and E^1^_2g_(Γ) modes, and an enhancement in the intensity. Notably, at an irradiation intensity of 400 Gy, a phase transition from antiferromagnetic to ferromagnetic behavior was observed, underscoring the profound impact of γ-radiation on the magnetic properties of the WS_2_ monolayer.

When a high-energy electron interacts with the nucleus in the target, only a small fraction of the incident electron energy can be transferred to the nucleus due to the conservation of momentum. In this way, high electron energies (greater than or equal to ‘threshold energies’) are required to displace an atom by electron-nucleus scattering.^23^ For instance, to permanently displace an atom in a graphite structure, about 20 eV of energy must be transferred to a carbon atom with an electron energy of 100 keV. Conversely, electron–electron scattering has the potential to induce ionization or bond breaking even at low electron energies. Although this energy transfer typically doesn't lead to atomic displacement, the localized reaction can still cause damage to the targeted materials. The cross-sections for nuclear and electron scattering decrease as electron energy increases. However, observable electron-nuclear scattering effects only occur at energies above the displacement threshold. Thus, the modification of catalysts with electron beams usually requires high energies, and the concentration of defects can be modulated by adjusting different energies and doses. As a result, a comprehensive understanding of these interactions, along with the threshold energies required for atomic displacements, is essential for controlling and optimizing the performance of catalysts at the nanoscale.

Defects induced by high-energy irradiation play a crucial role in expanding the surface area of catalysts, therefore facilitating the greater absorption of pollutants. For example, Sun *et al.*^76^ successfully enhanced the photocatalytic activity of Ag/Bi_2_WO_6_/CdWO_4_ through electron beam irradiation treatment. The intentional irradiation treatment not only induced interface defects but also facilitated the migration of catalytically active substances to the surface of the catalyst, leading to an increase in the content of free hydroxyl groups and significantly boosting the photocatalytic activity. Zhang *et al.*^77^ synthesized an FeNi_3_-16 catalyst with abundant defects by irradiating FeNi_3_ with H^+^ ions, which exhibited remarkable electrocatalytic activity towards the OER. The experimental results showed that the introduction of dual metal ion-induced defects activated initially inactive metal sites and significantly improved the OER catalytic performance compared to that of FeNi_3_. To further explore these effects, different defect models were constructed using DFT calculations, including iron defects (V_Fe_), nickel defects (V_Ni_), and iron and nickel defects (V_Fe_–_Ni_). The total density of states (TDOS) analysis revealed minimal variation in the electron occupation states. However, partial density of states (PDOS) for Fe and Ni atoms (Fig. 6b–d) showed significant differences. For Fe atoms, the distances between the d-band centers and the Fermi level are −2.47, −2.58, and −2.32 eV, while for Ni atoms, these distances are −1.57, −2.58, and −2.32 eV, respectively. The separation between levels is 1.57, 1.67, and 1.44 eV, respectively (Fig. 6e–g). These results suggest that H^+^ ion irradiation brings the d-band center closer to the Fermi level, due to the synergistic effects of the dual defects. This closer proximity enhances electron transfer and improves the binding affinity of adsorbed oxygen to metal ions, ultimately boosting OER catalytic activity.

γ-Ray irradiation is produced by the decay of the radioisotope ^60^Co, and the high-energy photons generated from irradiation have high penetration.^78,79^ The experimental conditions of γ-ray irradiation are feasible and straightforward, and it can be performed in the ambient environment. Thus, γ-ray irradiation can be performed with a large sample stage area of up to one square meter, making possible the development of catalysts with high efficiency, economy, mass production, and stable chemical properties. γ-Ray irradiation plays an important role in changing the surface structure and electronic properties of catalysts by interacting with matter through processes such as the photoelectric effect, Compton scattering, and the electron pair effect.^32,78^ In a recent study, Dong *et al.*^32^ used ^60^Co γ-ray irradiation to introduce defects in MoS_2_ to optimize HER performance. Interestingly, irradiation induced the generation of abundant sulfur (S) vacancies, which not only increased the number of active sites on the surface but also effectively tuned the intrinsic electronic structure of MoS_2_, thus accelerating reaction kinetics and ultimately enhancing the performance of the HER. It is noteworthy that excessive irradiation led to the passivation of S vacancies by oxygen atoms, which reduced the concentration of S vacancies and exhibited inferior electrocatalytic activity. Thus, the appropriate irradiation dose is crucial for enhancing the HER performance of MoS_2_ nanosheets. These findings highlight the potential of gamma-ray irradiation as a powerful tool to tailor the properties of catalyst materials and provide insights into the development of advanced highly functional photo-/electrocatalyst catalysts. Similarly, Chavda *et al.*^80^ investigated the effect of γ-ray irradiation on the physical properties of the MoS_2_ monolayer. The DFT calculations indicate that the bandgap of MoS_2_ decreases as the gamma irradiation dose increases, and the conductivity qualitatively increases due to the creation of additional defect states.

In addition, proton beam irradiation has been increasingly utilized to introduce vacancies into the material. For example, Choi *et al.*^40^ found that the ORR performance of the MnO_2_ catalyst can be effectively boosted by high-energy proton particles (14 MeV). The research found the material surface had produced abundant oxygen vacancies through the radioactive decomposition of water along the proton beam. These irradiation-induced oxygen vacancies create additional levels in the conduction or valence band, narrowing the band gap of MnO_2_. This narrow band gap enhances the electronic conductivity of oxygen-deficient MnO_2_, allowing easier electron transfer between MnO_2_ and oxygen molecules.

#### 3.2.2 Phase transformation

Phase transformation engineering has garnered significant attention due to its critical roles in modulating the crystal structure of catalysts to enhance catalytic activity. However, achieving desired crystalline phases or precisely controlled phase transitions at the nanoscale is challenging with conventional hydrothermal methods. In contrast, irradiation provides precise control over crystal phase transitions due to its nanoscale resolution and adjustable energy and dose parameters. Under optimal conditions, irradiation technology enables the tuning of catalyst structures and induces phase transformations, significantly altering their chemical and physical properties for better catalytic performance.

Recently, Dong *et al.*^81^ investigated the enhancement of HER performance in MoS_2_ nanosheets through phase transition engineering *via* 1 MeV e-beam irradiation. Following irradiation, the Raman spectra of MoS_2_ nanosheets revealed new characteristic peaks, indicating the formation of the 1T-MoS_2_ phase. Additionally, Fourier transform (FT) analysis of the irradiated samples showed a notable decrease in the Mo–Mo bond length, confirming the phase transition. With increasing electron beam fluence, a higher proportion of 1T phases was observed, activating additional electrocatalytic sites on the inert planes, resulting in better HER performance. Yue *et al.*^82^ employed 1 MeV electron beam irradiation to convert 2H–WSe_2_ to 1T-WSe_2_, which induced new characteristic peaks for the 1T phase of W at 31.5 and 33.6 eV, corresponding to the W4f_5/2_ and W4f_7/2_ states in WSe_2_. Additionally, new selenium peaks emerged at 54.6 and 55 eV, with binding energies for Se3d_5/2_ and Se3d_3/2_ measured at 5 eV. These changes in binding energy indicated alterations in lattice symmetry due to the irradiation. As irradiation fluence increased, the crystalline phase of WSe_2_ transitioned from 2H to 1T. The 1T-WSe_2_ structure not only activates previously inert basal planes and increases the total number of active sites but also improves electrical conductivity, thereby enhancing electron transport efficiency and exhibiting enhanced HER performance. Similarly, Jouini *et al.*^83^ utilized γ-ray irradiation to induce a phase change from NiO to Ni_2_O_3_. Fluorescence quenching behavior indicated the degradation of NiO and its transformation to Ni_2_O_3_.

#### 3.2.3 Morphology control

The morphology of a catalyst influences not only its surface area but also the exposure of active sites, diffusion pathways, and the overall reactivity properties. By adjusting parameters such as particle beam density, energy and irradiation time, the chemical composition and morphology of the catalyst surface can be precisely controlled. Recently, Kozlovskiy *et al.*^84^ investigated the structure and morphology of TiO_2_ thin films with O^2+^ ion radiation with an irradiation dose of 10^14^–10^16^ ions per cm^2^. The irradiation mechanism can be described as follows: the low energies of the incident ions lead to the approximately equal probabilities of elastic interactions and inelastic interactions. Consequently, the probabilities of point defect formation and initial atomic breakdown are also equal. The transfer of energy from the incident nucleus to the target nucleus is so strong that it can easily break chemical and crystal bonds. As a result, the oxygen atom is displaced from its equilibrium position due to its lighter mass compared to the titanium atom. Once the oxygen atom deviates from its equilibrium position, irradiation-implanted oxygen ions with high residual energy progressively fill cavities and gaps as they move across the surface of nanostructures, which distorts the crystal structure. In addition, the electrons generated by the collision form a secondary breakdown electron cascade, exacerbating the structural distortion. The results show that high doses of irradiation resulted in the appearance of mound-like and wavy inclusions in the films. Due to the magnetron sputtering of the film formation process, the initially synthesised films contain a small number of defects and dislocations. However, during the irradiation process, the energy transferred to the target atoms greatly exceeds the binding energy of the target nucleus, resulting in the lighter atoms being impacted out of the target nucleus and detached from their lattice sites, and the lattice being deformed as well. An orthogonal lattice structure characterised by three crystal axes leads to anisotropic deformation and the formation of defects and hollow regions. These regions can be filled with implanted oxygen atoms. Furthermore, the deformation and distortion of the crystal structure leads to changes in the lattice volume, and some of the deformed regions are squeezed towards the grain boundaries, which are the source of defects. The modification of thin films by ion beam irradiation will lead to the change of grain orientation and further change of morphology, which is beneficial for us to reasonably regulate the morphology of catalyst materials to obtain the ideal catalytic performance.

In particular, helium implantation has some advantages in the modification of the shape of the material. As a light element, it can cause little damage to catalysts, and it is easy to form gas bubbles. Recently, Liu and his colleagues^85^ have developed porous TiO_2_ nanorod array photoelectrodes by using He^+^ ion implantation to enhance photoelectrochemical water-splitting. Generally, when He atoms are implanted into TiO_2_ nanorod arrays, their insolubility in water allows them to be easily trapped by defects, forming bubbles as the fluence is increased or the temperature is raised. The study revealed that the size of the bubbles increased as the irradiation fluence increased. Additionally, annealing the sample in air at 500 °C resulted in larger bubbles and a more uniform distribution. The annealing process allowed the bubbles to absorb other dispersed helium atoms and vacancies. At high enough annealing temperatures, some He atoms escape from the bubbles and form nanoholes. This process generates a large number of nanoholes in the implantation layer of TiO_2_ nanorod arrays (NRAs). The formation of nanoholes effectively improves the hole trapping rate and charge carrier separation efficiency, and inhibits recombination, which therefore improves the photo-/electrocatalytic performance. Similarly, the combination of He^+^ ion implantation and post-annealing methods to regulate the morphology of α-Fe_2_O_3_ for enhanced photoelectrochemical performance has also been investigated by Wu *et al.*^86^ As the irradiation increases, a significant number of nanocavities are formed inside the He^+^ ion-implanted α-Fe_2_O_3_ NRAs. Relevant studies have shown that reasonable control of the parameters of helium ion irradiation fluence and thermal annealing can modulate the catalyst morphology to obtain more efficient catalytic performance.

#### 3.2.4 Element doping

High-energy irradiation offers a pollution-free approach with precise control over impurity distribution, enabling high-purity doping with excellent uniformity.^10^ Functionalization of catalysts by doping heteroatoms through irradiation techniques shows significant promise.^87^ Notably, medium-energy ion beams and high-energy γ-ray irradiation can penetrate the surface from several hundred nanometers to micron-scale depths, facilitating modifications in various nanostructured catalysts.^21^ Recently, high-energy ionizing irradiation technology has been used to dope heteroatoms into TiO_2_, WS_2_, NiO and graphene to boost their photo-/electrocatalytic activity. Specifically, WS_2_ is a promising electrocatalyst, but still faces serious challenges due to its poor electron transfer ability, lack of available active sites and poor long-term stability.^88–90^ In order to overcome these inherent drawbacks and further enhance the HER activity, numerous studies have been devoted to the improvement of electrical conductivity and the generation of more active sites.^91–93^ Among them, doping Fe ions is a promising strategy for tuning its electronic structure and modulating the hydrogen adsorption energy on its surface. Typically, the introduction of Fe ions is achieved through ordinary hydrothermal methods, which face many problems such as uneven doping, long reaction time, and low success rate.^91,94^ Recently, Cao *et al.*^95^ reported that Fe^13+^ ions were successfully introduced into WS_2_ after irradiation under the fluence of 8 × 10^13^ and 5 × 10^14^ ions per cm^2^ followed by annealing treatment. Within a few picoseconds, Fe^13+^ ion irradiation created multiple vacancies in WS_2_, enabling the incorporation of Fe ions. The XPS results confirmed the existence of Fe^2+^ and Fe^3+^ in the sample. This catalyst exhibited superior HER electrocatalytic activity in alkaline medium after irradiation, and the improved HER activity was attributed to the exposure of more active sites on the inert basal plane of WS_2_ and the improved electrical conductivity due to the Fe-doping, which thereby reduced the resistance to charge transfer, and accelerated the electron transfer.

**Fig. 7:**
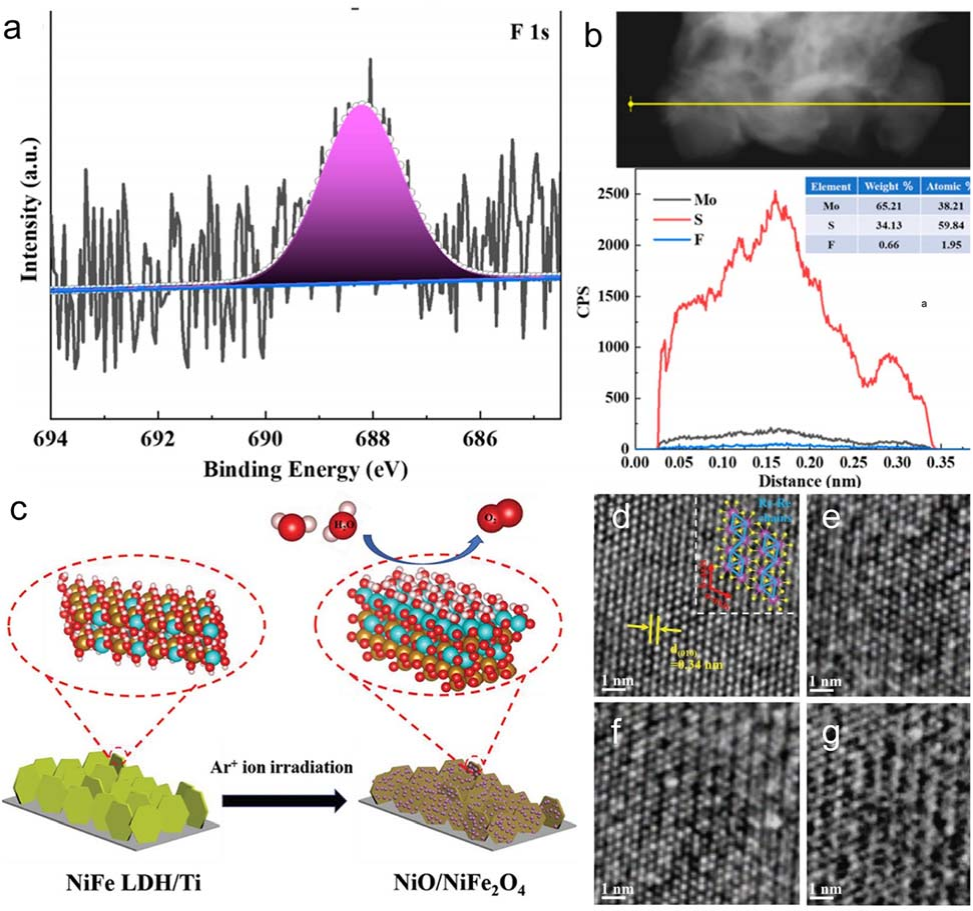
(a) XPS spectra of F 1s from irradiated MoS_2_ and (b) line-scan profiling analysis of irradiated MoS_2_. Copyright 2018, American Chemical Society. (c) Schematic illustration of the formation of NiO/NiFe_2_O_4_. Copyright 2021, Wiley-VCH. (d) HRTEM images of pristine ReS_2_ nanosheets with observation direction [001], (e) Ar^+^ ion beam irradiation of ReS_2_ nanosheets for 10 s, (f) 30 s, and (g) 60 s.^74,84,96,97^ Copyright 2018, Wiley-VCH.

Dong *et al.*^74^ doped F ions into MoS_2_ by heavy-ion irradiation, achieving successful doping through XPS analysis. The chemical compositions and valence states of MoS_2_ NSs before and after irradiation are shown in Fig. 7a. Generally, the F 1s peak is located at ∼688 eV in the XPS spectrum. However, F 1s does not show a significant peak in the XPS spectrum of F-2 × 10^13^. This is because the XPS penetration depth is less than ∼10 nm, which limits the detection of the sample surface. Line-scan profiling in Fig. 7b further confirms successful F doping in MoS_2_. All of this suggests that ion beam irradiation serves as a novel and effective method for elemental doping.

γ-Ray irradiation is a promising, convenient and environmentally friendly method for the production of graphene-based nanomaterials. It also contributes to the reduction of metal ions and the modification of the carbon properties and the chemical reactions on the surface. Recently, Rahman *et al.*^98^ have synthesized a N-doped RGO-supported Fe-based catalyst with 100 kGy γ-ray irradiation, and the percentage by weight (wt%) of Fe loading is in the range of 10% to 20%. XRD, Raman, FTIR, and EDS characterizations revealed that the γ-ray radiation effectively introduced N and Fe into the sample. The N-doping increases the electron density and forms N-support sites with carbon and iron, which directly improved its ORR performance. Additionally, the strong interactions between Fe-N_*x*_ and O_2_ facilitated rapid electron transfer.

Recently, Ghicov *et al.*^99^ investigated the effects of N ion implantation on the structure and doping efficiency of TiO_2_ nanotubes, using ion fluences of 1 × 10^15^ and 1 × 10^16^ ions per cm^2^ under 60 keV acceleration energy. At a high fluence of 1 × 10^16^ ions per cm^2^, the ion implantation disrupts the original morphology of the self-organized TiO_2_ nanotubes, resulting in complete amorphization of the anatase structure. This amorphous state, however, created traps for photogenerated electron–hole pairs, leading to reduced photocurrents in the UV range. Interestingly, despite these structural alterations, the specific dose of 1 × 10^16^ ions per cm^2^ enabled effective N doping, which enhanced the photo-response in the visible range.

Different forms of heteroatom doping play distinct roles in enhancing catalyst properties, necessitating the strategic selection of ions for catalyst irradiation. Recently, Liu *et al.*^100^ investigated the effects of noble metal nanoparticles and metal (Fe, V) and non-metal (N) doped TiO_2_, using energies from 20–50 kV and fluences ranging from 0.1–20 × 10^16^ ions per cm^2^. After ion implantation and annealing treatments, the photocatalytic activity of the Au–TiO_2_ samples was enhanced, which was attributed to the increased absorption of incident light by Au nanoparticles, enhanced local electric field and reactive electron and hole excitation. Additionally, a Schottky junction also formed when Au nanoparticles made direct contact with the TiO_2_, significantly promoting electron–hole separation and reducing recombination. For Fe-doped TiO_2_, the mixing of Ti^4+^ 3d and Fe^3+^ 3d orbitals introduced new energy levels into the TiO_2_ band structure, increasing visible light responsiveness. Meanwhile, V doping accelerated the phase transition of TiO_2_ from anatase to rutile. In the case of N-doped TiO_2,_ experimental and theoretical analyses demonstrated that replacing oxygen with nitrogen created isolated N 2p localized states above the O 2p-dominant valence band. The mixing of N 2p and O 2p states reduced the TiO_2_ band gap, enhancing visible light absorption. Overall, TiO_2_ thin films prepared *via* ion irradiation with Au, Fe, V, and N were highly crystalline, stable, and showed improved photocatalytic activity under visible light. These promising results underscore that different ion sources have varying effects on the structural and physicochemical properties of catalysts, emphasizing the importance of tailored doping strategies.

#### 3.2.5 Band gap engineering

Generally, the ideal photocatalyst should have a band gap of 1.8–2.3 eV, which allows for effective visible light absorption and generates sufficient photogenerated charge carriers.^101^ However, many common photocatalysts have larger band gaps, such as ZnO, SnO_2_ and TiO_2_ (3.2 eV), limiting their visible light utilization. To address this, regulating the band gap of these semiconductor photocatalysts can improve their optical and electrical properties for better performance. In particular, doping metal ions is a widely employed tactic for introducing impurity levels into the forbidden band gap, thereby narrowing the band gap to facilitate the absorption of visible light. The impurity levels are strategically positioned above the valence band or below the conduction band, functioning as acceptor or donor levels, respectively. Specifically, the presence of acceptor levels and donor levels affects key properties of semiconductors such as light absorption, carrier lifetime, and photoconductivity. The acceptor level is located near the conduction band, where it traps electrons in the conduction band, thereby affecting the electron concentration and conductivity of the semiconductor. Meanwhile, donor levels are located near the valence band, which provides additional free electrons and increases the conductivity of the semiconductor.

There are many methods for doping metal ions into wide bandgap materials, such as co-precipitation synthesis and advanced ion implantation.^102–105^ Unlike the conventional chemical methods that yield unstable metal ion doping, advanced physical doping methods (*e.g.*, ion beam irradiation) can effectively dope metal ions into catalysts in a controlled manner.^106^ Specifically, ion irradiation not only facilitates metal ion doping but also alters the electronic and surface properties of catalysts, making it particularly effective for doping. In a recent work, hydrothermal growth of ZnO nanorod arrays on fluorine-doped tin oxide (FTO) substrates was reported by Wang *et al.*, followed by doping with varying fluxes (3 × 10^15^, 5 × 10^15^, and 2 × 10^16^ ions per cm^2^) of Cu ions using ion implantation techniques.^107^ The doping of Cu ions within the forbidden band gap notably reduces the band gap of ZnO, attributable to the introduction of impurities in the form of Cu^2+^ and Cu^+^ states. Electrons in the valence band can be excited to additional copper doping levels and subsequently enter the conduction band of ZnO when exposed to visible light, thereby expanding the light absorption range of the Cu-doped ZnO nanorods. Similarly, Cai *et al.*^108^ successfully prepared ZnO NRs doped with V ions by an ion implantation method (energy of 50 keV, flux of 2.5 × 10^14^–5 × 10^15^ ions per cm^2^). The introduction of impurity donor levels through the doping of V^4+^ ions within the forbidden band has been observed to notably reduce the band gap of ZnO, thereby extending the light absorption of ZnO NRs into the visible region. As a result, the V-ion doped ZnO NRs exhibited superior photocatalytic activity to pristine ZnO. Under visible light irradiation, the V-ion doped ZnO NRs demonstrated the ability to excite additional electrons from the V^4+^ dopant into the conduction band of ZnO. Furthermore, as the V ion implantation dose increased, so did the charge carrier density, leading to the generation of more electrons and holes for water oxidation reactions.

#### 3.2.6 Heterostructure formation

The creation of heterojunctions in a hybrid nanosystem can effectively enhance the catalytic performance of catalysts.^109^ The heterogeneous structure not only expands the electrode surface area, but also increases the contact area of the reactants on the catalyst surface, thereby achieving high catalytic activity. Ionizing irradiation facilitates the formation of distinct heterogeneous structures due to its controllability and the non-uniform distribution of energy.^110,111^ In particular, ion beam irritation offers a precise method for adjusting the electronic structure of a catalyst by promoting the formation of two distinct nanostructures. For example, if the impact caused by the implanted ions exceeds the solid solubility of the material, these ions will disperse and aggregate on the surface of the material, resulting in the formation of NPs.^112^ By carefully controlling the implantation-precipitation process, it is possible to create heterostructures within the catalyst, which have the potential to significantly enhance the intrinsic activity of the catalyst. Recently, Zhong *et al.*^96^ have successfully fabricated a NiO/NiFe_2_O_4_ heterostructure by the controlled irradiation of NiFe layered double hydroxide (LDH) nanosheets with Ar^+^ ions (Fig. 7c). During irradiation, the incident Ar^+^ ions undergo a series of collisions with NiFe LDH atoms. When the energy of the injected Ar^+^ ions exceeds the displacement energy, NiFe LDH atoms are ejected from the lattice and a large number of atomic voids are formed. Due to the lower displacement energy, O and H atoms are more easily ejected from the lattice. As the fluence increases, more and more H and O atoms are displaced, leading to the transformation of NiFe LDH into NiO and amorphous NiFe_2_O_4_. The DFT calculations revealed that this heterogeneous structure effectively reduced the energy barrier for the O^*^ to OOH^*^ transition. The synergistic effect of NiO and NiFe_2_O_4_ enhances the reaction kinetics and accelerates the charge transfer as well as increasing the active site exposure, thereby exhibited better OER performance.

## 4 Catalytic applications

### 4.1 Electrocatalysis

Electrochemical water splitting for hydrogen production is a highly promising strategy for sustainable energy supply, offering a clean alternative to reduce reliance on fossil fuels. However, the inherent slow kinetics of the HER and OER necessitate the use of highly active catalysts.^113,114^ Noble metals, such as Pt and IrO_2_, exhibit excellent electrocatalytic activity, but their scarcity and high cost pose significant barriers to large-scale application. Transition metal-based compounds, such as those containing, *e.g.*, Fe, Co, Ni, Mo and W, offer a promising alternative due to their abundance and easy synthesis. However, optimizing these materials to achieve outstanding catalytic activity and high stability for practical applications remains a major challenge. In this context, ionizing and non-ionizing radiation techniques offer unique merits for the development of catalysts toward water splitting (Table 2). By creating atomic-scale defects, activating surface sites, and altering the electronic structure of the catalysts, radiation can significantly enhance their intrinsic electrocatalytic activity.

**Table 2:** Summary of the applications of various types of irradiation technology in synthesis/modification of electrocatalysts from recent work

Catalyst materials	Irradiation sources	Energy	Fluence	Roles	Application	Ref.
Thin-layer MoS_2_	γ-Ray irradiation/^60^Co	—	100 rad s^−1^ (0.1, 1, 10, 100 Mrad)	Defect introduction	HER	32
MoS_2_ nanosheets	Electron beam irradiation	1 MeV	5 × 10^14^, 5 × 10^15^, 1 × 10^16^ e cm^−2^	Defect introduction and phase transformation	HER	81
Bulk MoS_2_	Ion beam irradiation/S, Se, and Te	400 keV	1 × 10^14^–1 × 10^16^ ions per cm^2^	Doping and vacancy introduction	HER	69
Layer MoS_2_	Swift heavy ion irradiation/Xe	91 MeV	5 × 10^12^, 2 × 10^13^ and 5 × 10^13^ ions per cm^2^	Vacancy introduction	HER	94
MoS_2_	Ion beam irradiation/C^+^	3 MeV	5 × 10^12^, 2 × 10^13^ and 5 × 10^13^ ions per cm^2^	Defect engineering	HER	115
WS_2_	Ion beam irradiation/Fe^13+^	—	8 × 10^13^ and 5 × 10^14^ ions per cm^2^	Fe-doping and vacancy introduction	HER	95
Bi_2_Te_3_ nanosheets	Ion beam irradiation/Fe^10+^	320 keV	4 × 10^14^ ions per cm^2^	Defect introduction and surface modification	HER	116
Sb_2_Te_3_/Ti	Ion beam irradiation/N^+^	15 keV	2 × 10^14^, 2 × 10^15^, and 2 × 10^16^ ions per cm^2^	Doping and defect introduction	HER	71
Si	Ion beam irradiation/Ar^+^	90 keV	5 ×10^16^–2 × 10^17^ ions per cm^2^	Morphology control	HER	117
MoSe_2_ nanosheets	Ion beam irradiation/Ar^2+^	—	5 × 10 ^14^,5 × 10 ^15^ ions per cm^2^	Vacancy introduction	HER	118
WSe_2_	Electron beam irradiation	1 MeV	1 × 10^13^, 1 × 10^14^, 5 × 10^14^, 1 × 10^15^, 1 × 10^16^ e cm^−2^	Phase transformation	HER	82
Ti_3_C_2_T_*x*_ mxene	γ-ray irradiation/Core fuel bundles	—	500 Gy h^−1^ (100, 300 kGy)	Morphology control	HER	119
12- Tungstophosphoric acid	Ion beam irradiation/C^+^	10 keV	5 × 10^14^–2.5 × 10^15^ ions per cm^2^	Structural modification	HER	120
Single-layer MoS_2_	Ion beam irradiation/Au	500 keV	5 × 10^11^–1 × 10^14^ ions per cm^2^	Defect engineering	HER	28
CoO/Co_3_O_4_	Ion beam irradiation/Ar^+^	50 keV	5 × 10^15^ ions per cm^2^	Morphology control/phase transformation	OER	70
Co_3_O_4_ -Ov	Ion beam irradiation/Ar^+^	25 keV	5 × 10^15^ ions per cm^2^	Oxygen vacancy introduction	OER	70
NiO/CC	Ion beam irradiation/N^+^	50 keV	5 × 10^15^ ions per cm^2^	N-doping and vacancy introduction	OER	121
NiO/NiFe_2_O_4_	Ion beam irradiation/Ar^+^	100 keV	1, 10, 20, 30, 40 × 10^15^ ions per cm^2^	Heterostructure formation	OER	96
Pt/N_50_-GY	γ-Ray irradiation/^60^Co	—	140 kGy	Morphology control	ORR	47
Pt-Ru/HOPG	Ion beam irradiation/N	100 eV	4 × 10^15^–9.6 × 10^16^ ions per cm^2^	Doping and defect	ORR	166
Fe/N-rGO	γ-Ray irradiation/^60^Co	—	100 kGy	Fe/N-doping	ORR	98
CN/Pt, g-C_3_N_4_, Pt	γ-Ray irradiation	—	20, 60, 100, 140, 180 kGy	Material synthesis	ORR	167
Pt_0.9nm_/Pt_25_Ni_75_(111)	Ion beam irradiation/N_2_^+^, N^0^_2_	≤100 eV	—	Material synthesis	ORR	168
Pt-CeO_*x*_ nanowire/C	Proton irradiation	—	5 kGy	Defect introduction	ORR	41
MnO_2_	Proton irradiation	14 MeV	—	Vacancy introduction	ORR	40
Pd/CNT	γ-Ray irradiation/^60^Co	—	15, 25, 50, 100 kGy	Material synthesis	ORR	169
PRGO	γ-Ray irradiation/^60^Co	1.33 MeV	15, 25 kGy	Vacancy introduction	ORR	129
Pt NPs/GC	Ion beam irradiation/Ar	380 kev	7.5 × 10^15^ ions per cm^2^	Defect introduction	ORR	128
Pt/CN-CB	γ-ray irradiation/^60^Co	—	140 kGy	Material synthesis	ORR	170
Pt/HOPG	Ion beam irradiation/Ar^+^	1 keV	1.0 × 10^14^ ions per cm^2^	Vacancy introduction	ORR	171
Pt/HOPG	Ion beam irradiation/Ar	100 eV	∼5 × 10^16^ ions per cm^2^	Defect engineering	ORR	172
Pt/HOPG	Ion beam irradiation/N	100 eV	∼5 × 10^16^ ions per cm^2^	N-doping	ORR	173
Pt–Ru/HOPG	Ion beam irradiation/Ar	100 eV	4 × 10^15^–9.6 × 10^16^ ions per cm^2^	Doping and defect	ORR	174
PtPb nanoplates	Ion beam irradiation/C^+^	10 eV	1 × 10^16^, 2 × 10^16^, 3 × 10^16^ ions per cm^2^	Defect engineering and interface engineering	ORR	72,73
GY-PtPd	γ-ray irradiation	—	150 kGy	Material synthesis	ORR	175
Fe–N–C	Electron beam irradiation	10 eV	80 kGy	Forming Fe–N_*x*_ active site	ORR	176
PtNPs/NrGO	γ-Ray irradiation/^60^Co	—	100 kGy	Morphology control	ORR	177

#### 4.1.1 Hydrogen evolution reaction (HER)

Recently, two-dimensional layered chalcogenides, such as MoS_2_, WS_2_ and MoSe_2_, have emerged as a promising, cost-effective alternative to Pt.^122,123^ Nevertheless, the catalytically active sites of these layered chalcogenides are primarily located at the edge sites, whereas the basal plane remains largely inert toward the HER.^124^ Given that the surface area of the basal plane of 2D layered chalcogenides significantly surpasses that of edges, introducing defects into the basal plane can effectively improve the number of active sites for better performance. However, it's a challenge to achieve controlled defect introduction into the basal plane. Coincidentally, ionizing irradiation offers several advantages for introducing defects into the basal plane of MoS_2_. Firstly, ionizing irradiation operates on a high-energy and momentum-driven basis, facilitating a swift and efficient introduction of defects into the basal plane, enabling a time-efficient and scalable process. Secondly, ionizing irradiation can precisely tailor the number and distribution of defects in the basal plane by adjusting the irradiation parameters, which allows for a precise modulation of electrocatalytic behaviours. Furthermore, ionizing radiation is a non-chemical and impurity-free strategy, ensuring that the introduced defects do not compromise the structural integrity of the material, maintaining the favourable electronic structure.

Recently, Xia *et al.*^118^ have synthesized defective MoSe_2_ nanosheets on carbon cloth by a CVD method and it was found that the intentional irradiation with high energy Ar^2+^ ions (5 × 10^14^ and 5 × 10^15^ ions per cm^2^, respectively) had induced multifold in the basal plane. Four types of vacancy were observed in their work: native Mo and Se vacancy (V_Mo_ and V_Se_), double Se vacancy (V_Se2_), and the absence of one MoSe_2_ (V_MoSe2_). As expected, the irradiated MoSe_2_ with a dose of 5 × 10^15^ ions per cm^2^ displayed a much lower overpotential and Tafel slope than those of pure MoSe_2_. The improved HER performance can be attributed to the increased catalytic sites and optimized electronic structure due to the formed vacancies. Sun *et al.*^115^ utilized C^+^ ions to irradiate MoS_2_ with an energy of 3 MeV at various ion fluences (5 × 10^12^, 2 × 10^13^, and 5 × 10^13^ ions per cm^2^). The ion irradiation activated the inert basal plane of MoS_2_ NSs by introducing numerous S vacancies, which thereby exhibited much improved HER performance. Additionally, it was found that different ion fluences could modulate the number of S vacancies and amorphous phases on the MoS_2_ substrate. In particular, the MoS_2_ nanosheets irradiated at an ion fluence of 2 × 10^13^ ions per cm^2^ exhibited the best HER performance with an onset potential of 77 mV and a Tafel slope of 66 mV dec^−1^. In addition, the ion species, energy, fluence, and other irradiation parameters has distinct effects on vacancy formation within the catalyst. Madauss *et al.*^94^ reported a new method to improve the HER performance of MoS_2_ by using a rapid heavy Xe ion irradiation (irradiation energy 91 MeV). At −0.6 V *vs.* RHE, the irradiated MoS_2_ sample exhibited a higher current density (35.3 mA cm^−2^) than pristine MoS_2_ (−13.3 mA cm^−2^). The irradiated samples also had a smaller Tafel slope (104 mV dec^−1^) compared to pristine MoS_2_ (106 mV dec^−1^). He *et al.*^28^ fabricated a single layer of MoS_2_ using 500 keV high-energy Au-ion irradiation. They found that ion irradiation primarily generated S vacancies. As the defect density increases, the photoluminescence (PL) peak first blueshifts and then redshifts due to the electron transfer from MoS_2_ to the adsorbed O_2_ at the defect sites.

γ-ray radiation serves as a source of high-energy photons with strong penetrating ability, allowing strong penetration through the sample and ensuring a more uniform irradiation effect. In a recent study, Dong *et al.*^32^ employed ^60^Co γ-ray irradiation to modify MoS_2_ nanosheets at doses of 0.1, 1, 10, and 100 Mrad to investigate the effect of irradiation fluence on the electrocatalytic performance. A series of characterizations showed that numerous S vacancies were produced on the MoS_2_ nanosheets after irradiation. Electrochemical measurements showed that at a dose of 10 Mrad, MoS_2_ nanosheets achieved a low overpotential (*η*_10_) value of 269.4 mV to achieve a current density of 10 mA cm^−2^ with a Tafel slope of 65.96 mV dec^−1^, which was much smaller than that of bare MoS_2_ (327.4 mV, 130.88 mV dec^−1^) and the irradiated samples with 100 krad (121.23 mV dec^−1^), 1 Mrad (74.97 mV dec^−1^) and 100 Mrad (74.97 mV dec^−1^) of MoS_2_, respectively. The above results indicated that the introduction of S vacancies increased the number of active sites on the MoS_2_ nanosheets, resulting in better HER performance. Subsequently, the same research group employed 1 MeV electron irradiation to irradiate MoS_2_ nanosheets, discovering that, besides forming S vacancies, it also induced a transition from the 2-H phase to the 1-T phase.^81^ This transition can be attributed to the charge redistribution associated with electronic excitation, the formation of S vacancies, and the accumulation of mechanical strain in defective MoS_2_ nanosheets. The formation of S vacancies and the 1T phase transition during irradiation greatly enhanced the HER activity. In particular, when the irradiation fluence reaches 5 × 10^14^ e cm^−2^, a current density of 10 mA cm^−2^ can be achieved with an initial overpotential of only 141 mV and an overpotential of 235 mV.^111^ Similarly, Li *et al.*^82^ achieved the conversion of 2H-phase tungsten selenide (2H–WSe_2_) to 1T-phase tungsten selenide (1T-WSe_2_) under 1 MeV electron irradiation. At an irradiation fluence of 5 × 10^14^ e cm^−2^, the minimum onset overpotential of WSe_2_ was 247 mV (*vs.* RHE), much lower than that of the as-prepared WSe_2_ (348 mV.) The Tafel slope of 1T-WSe_2_ was 67 mV dec^−1^, which was also lower than that of unirradiated WSe_2_ (117 mV dec^−1^). The enhanced electrocatalytic performance of WSe_2_ under electron beam irradiation is attributed to the presence of 1T-WSe_2_ components in properly irradiated samples. However, it's important to note that further increases in irradiation fluence lead to agglomeration of WSe_2_, which severely hindered the exposure of catalytically active sites and resulted in the decrease of HER catalytic activity.^92^

More recently, in an innovative work by Huang *et al.*,^97^ Re vacancy defects were intentionally introduced in ReS_2_ by a controlled Ar ion beam bombardment. The presence of Re vacancies not only generates enough unsaturated bonds but also affects the intrinsic charge compensation of the S–Re–Re bond, leading to the adsorption of H^+^ being neither too strong nor too weak. In addition, the vacancy densities increase with the increase of the ion beam intensity. When the irradiation time was extended to 60 s, a significant increase in lattice defects was observed, and most of the zigzag Re–Re chains were disrupted, which impeded the adsorption of H^+^ at the active site. The HRTEM images, as illustrated in Fig. 7d–g, demonstrate that Ar^+^ ion beam irradiation is conducive to the formation of Re vacancies with appropriate duration, and it is possible to efficiently control the vacancy density on 2D ReS_2_ nanosheets.

**Fig. 8:**
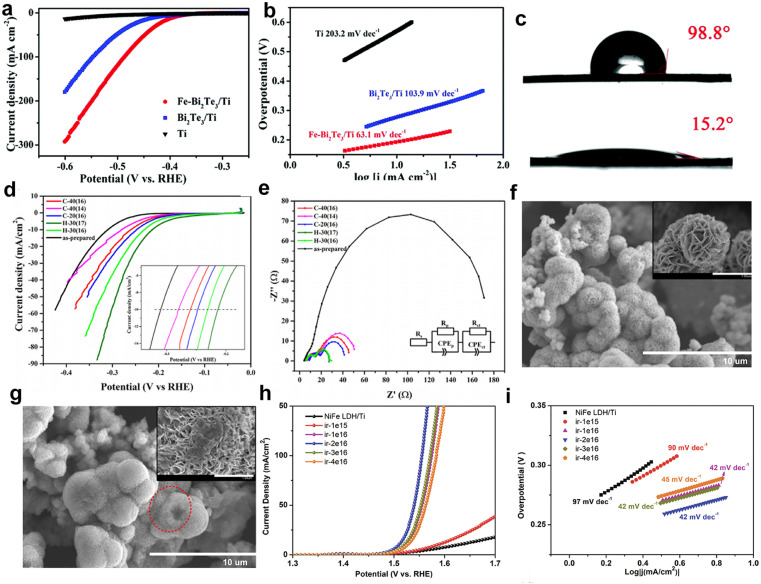
(a) LSV curves of Ti, Bi_2_Te_3_/Ti and Fe–Bi_2_Te_3_/Ti at a scan rate of 5 m V s^−1^. (b) Corresponding Tafel plots. (c) Contact angles for Bi_2_Te_3_/Ti (top) and Fe–Bi_2_Te_3_/Ti (bottom), respectively. Copyright 2020 The Royal Society of Chemistry. The HER properties of MoS_2_ (d); iR polarization curves; (e) EIS spectrum at an overpotential of 0.2 V *vs.* RHE; SEM images of as-prepared MoS_2_ (f) and irradiated sample C-40(16) (g); inset: magnified SEM image of as-prepared and irradiated MoS_2_. Copyright 2013 Elsevier Ltd. OER performance (h) and Tafel plots (i) of NiO/NiFe_2_O_4_ (ir-2e^16^), NiFe_2_O_4_, NiO, and RuO_2._^68,96,116^ Copyright 2021, Wiley-VCH.

The hydrophilic/hydrophobic properties have a significant impact on the electrocatalytic performance of catalysts. Enhancing the hydrophilicity of catalysts by various methods and strategies is an encouraging pursuit. Recently, Wang *et al.*^116^ investigated the effect of Fe^10+^ ion irradiation on the HER properties of Bi_2_Te_3_ nanosheets deposited on titanium plates. In acidic solution, the HER performance of Bi_2_Te_3_ nanosheets was significantly improved after irradiation with Fe^10+^ ions compared to that of pristine Bi_2_Te_3_ nanosheets, with *η*_10_ decreasing from 436 mV to 395 mV (Fig. 8a–c). The increase in activity can be attributed to the change of Bi_2_Te_3_ surface properties from hydrophobic to hydrophilic by irradiation with iron ions, which promotes the release of H_2_ bubbles on the catalyst surface and exposes the active sites in time. At the same time, it also prevents large gas bubbles from damaging the electrode, thus improving the stability of the catalyst. This efficient Fe^3+^ ion irradiation method provides an innovative approach for designing other efficient catalysts.

To enhance the intrinsic HER catalytic activity for flower-like MoS_2_, Mravik *et al.* irradiated MoS_2_ nanopowder with carbon and hydrogen ions.^68^ The HER performance of the irradiated samples was tested and the polarization curves of the prepared samples are shown in Fig. 8d. It was observed that all irradiated samples exhibited enhanced HER catalytic activity compared to bare MoS_2_, achieving an optimal *η*_10_ of 213 mV. A similar trend is observed for carbon ion irradiation, with an *η*_10_ of 259 mV for sample C-40 (16) and below 280 mV for sample C-40 (14). However, the impact of varying the energy of incident carbon ions was less pronounced.

Apparently, the HER performance of these catalysts was significantly enhanced after irradiation, resulting in a current density at 300 mV that was up to six times higher than that of the non-irradiated sample. Electrode kinetics were evaluated using EIS (Fig. 8e), revealing that the charge transfer resistance (*R*_ct_) of irradiated samples was considerably reduced, with hydrogen ion-irradiated samples showing an *R*_ct_ as low as 12 Ω, up to 14 times lower than that of unirradiated samples. Fig. 8f and g illustrate the morphology of the catalyst before and after irradiation. Initially, the sample displayed spherical flower-like structures composed of nanosheet petals (inset in Fig. 8f). After irradiation, the morphology was altered, with smoother spherical surfaces, disrupted flower-like structures, and partially “opened up” microspheres, as seen in the dashed circles in Fig. 8g, allowing easier access to the interior. Both carbon and hydrogen ion beams produced similar morphological changes. These findings suggest that ion irradiation induces structural defects and morphological changes, which enhance the conductivity and intrinsic activity of the catalysts, which significantly impacted their HER performance.

Wu *et al.*^117^ also reported the preparation of nanoporous silica (SiO_2_) NPs as HER catalysts by irradiation with 90 MeV Ar^+^ ions and annealing in a vacuum. The effect of fluence on cathode morphology and HER performance was investigated. The *η*_10_ of the samples irradiated at fluences of 5 × 10^16^, 1 × 10^17^, and 2 × 10^17^ ions cm^−2^ were 590, 357, and 483 mV, respectively, which were much smaller than that of the pristine silicon. The improvement in the HER performance was attributed to the formation of a porous structure by the irradiation of Ar^+^ ions, which facilitated the spillage of the H_2_ bubbles from the surface of the catalysts, exposing more active sites. The application of irradiation technology in the HER offers new perspectives in the energy fields, but many mechanisms remain to be explored.

#### 4.1.2 Oxygen evolution reaction (OER)

As one of the half-reactions in the water splitting process, the oxygen evolution reaction suffers from kinetic retardation due to its complex four-proton-electron transfer process.^125^ Despite extensive research on various transition metal compounds, there is still much space to improve their OER performance. Ionizing irradiation emerges as a potent tool for enhancing transition metal compounds with several obvious merits. Firstly, ionizing irradiation can introduce defects into materials, such as vacancies and interstitial sites, enhancing the number of active sites. Secondly, ionizing irradiation allows for the manipulation of crystal structures, inducing lattice distortions and phase transitions that optimize the exposure of active sites and formation of a heterostructure. Thirdly, the enhancement of electrical conductivity through ionizing irradiation plays a pivotal role in promoting efficient electron transfer during the OER process. Moreover, the ionizing irradiation treatment enhances the stability of catalysts, making the catalysts have stronger corrosion resistance and intrinsic activity during the OER process.

Numerous studies have showcased that irradiation can be employed as a distinctive method for engineering efficient and durable OER catalysts for overall water splitting. For example, Zhong *et al.*^96^ irradiated a NiFe LDH with Ar^+^ ions with various fluences. Benefiting from the formed oxygen vacancies and NiO/NiFe_2_O_4_ heterostructure, the catalyst with a fluence of 2 × 10^16^ ions per cm^2^ presented significantly improved OER performance with an *η*_10_ of 279 mV and a low Tafel slope of 42 mV dec^−1^, superior to that of NiFe LDH/Ti (Fig. 8h–j). Also, the DFT calculations have shown that the NiO/NiFe_2_O_4_ heterostructure has optimized the free energy of the oxygen-containing intermediates, thereby showing greater OER performance. Xia *et al.*^121^ prepared NiO nanosheets by a hydrothermal method following annealing treatment. Then, N^+^ ions were irradiated to introduce more oxygen vacancies and dope N into NiO, and the dose of N^+^ ion beam was 5 × 10^15^ ions per cm^2^. The roles of N-doping and O vacancies in the NiO substrate were examined by DFT calculation. The results of the density of states (DOS) showed that the bandgap of NiO decreased from 2.9 eV to 0.56 eV. Meanwhile, the charges are clustered around the O vacancy and N atoms, indicating that it has better electronic conductivity. Thus, the OER catalytic activity of NiO was much improved. In particular, the OER properties of N^+^-5 × 10^15^ (5 × 10^15^ N^+^ ions per cm^2^ irradiated samples) were superior to those of NiO/CC (2.32 V, 198.74 mV dec^−1^), and the potential is 1.98 V at 100 mA cm^−2^ and Tafel slope is 135.97 mV dec^−1^. Moreover, the high TOF value of N^+^-5 × 10^15^ (92.1 s^−1^) shows that the electron transfer rate is improved after N^+^ ion irradiation.

#### 4.1.3 Oxygen reduction reaction (ORR)

Fuel cells with high energy and power densities are regarded as ideal solutions for next-generation devices, standing out among the various energy conversion and storage systems developed to date. In the development of advanced fuel cells, maximizing the ORR activity of the Pt cathode while minimizing its Pt content is crucial. Transition metal oxides favor redox reactions due to their electronic properties.^126^ In addition, transition metals can exhibit a variety of oxidation states, thus facilitating electron transfer. Recently, Baeck *et al.*^40^ investigated the effect of a 14 MeV proton beam irradiation on the intrinsic ORR catalytic activity of MnO_2_. It was found that the proton irradiation introduced a large number of low-coordinate oxygen and defective sites, which provided multiple active sites and thus increased the ORR activity. Furthermore, the presence of oxygen vacancies introduced additional levels in the conduction or valence band, resulting in a narrower band gap. This narrower band gap enhanced the electronic conductivity of oxygen-deficient MnO_2_, as electrons can be easily transferred from MnO_2_ to O_2_. Additionally, the current density of proton-treated MnO_2_ reached −4.6 mA cm^−2^ at 0.65 V (*vs.* RHE). Therefore, the MnO_2_ treated by proton radiation exhibited remarkable durability, and the activity still remained at 95% after a 10 000 second time chronoamperometric stability test. Chauhan *et al.*^41^ fabricated Pt-CeO_*x*_/C (Pt/C = 0.02) using 5 kGy proton beam irradiation. Since the free radical density generated by the proton beam irradiation is twice as high as the electron beam irradiation, the surface of the CeO_*x*_ nanowire is completely transformed into a thin layer of Pt–O–Ce. As expected, the ORR performance is higher than that of the conventional Pt/C (Pt/C = 0.2) and the same as that for Pt-CeO_*x*_/C (Pt/C = 0.2). The characterization and surface atomistic simulations indicate that Pt–O–Ce bonds are formed in the defect-rich region of CeO_*x*_ nanowires, resulting in the maximum ORR activity of the prepared samples. The results suggest that the surface modification of CeO_*x*_ nanowires by proton beam irradiation can reduce the Pt content and maintain its high activity.

**Fig. 9:**
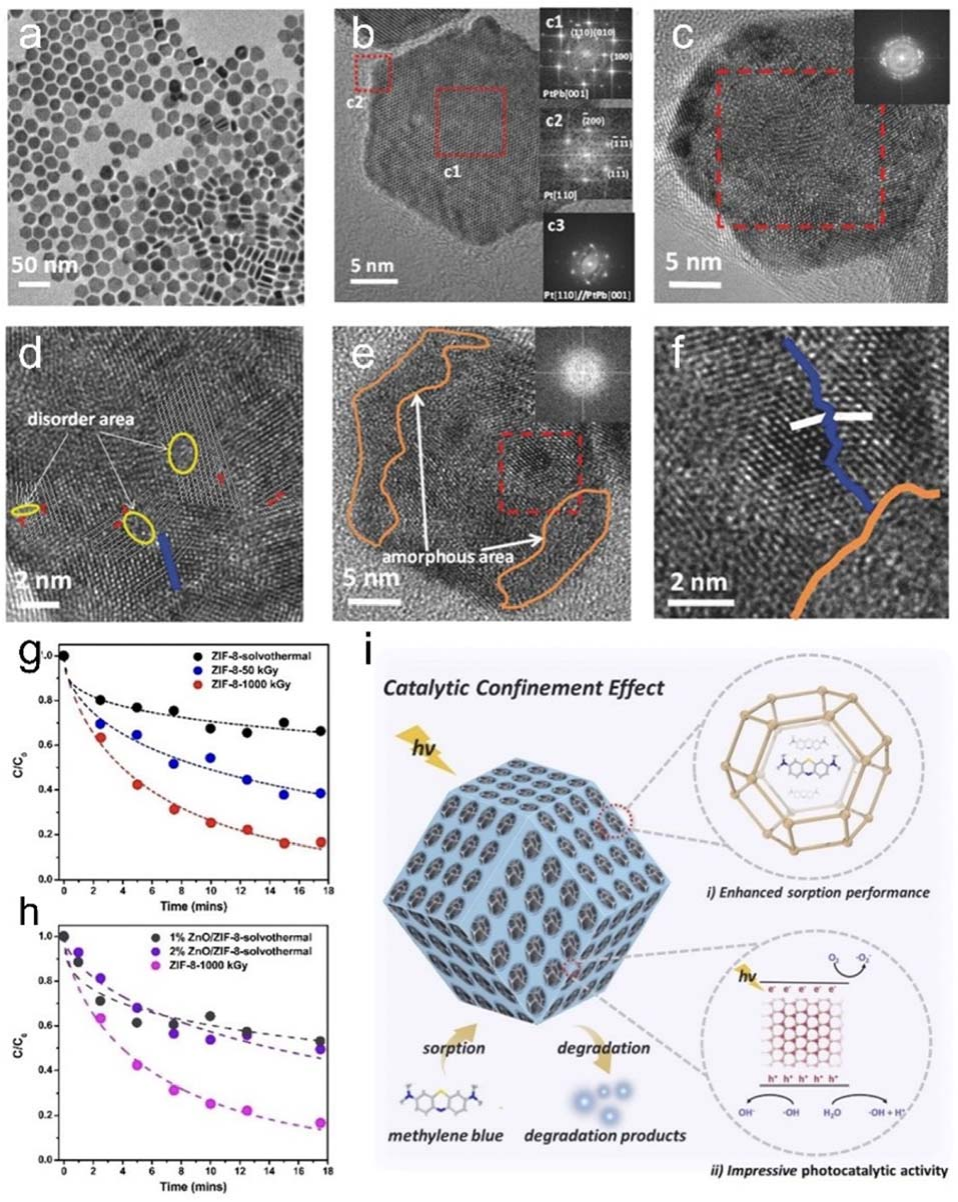
TEM (a) and HRTEM (b) images of PtPb nanosheets; (c–f) HRTEM image of PtPb/C after C^+^ irradiation. The inset in (c) is the FFT image of the whole nanosheet. The inset in (e) is the FFT image of the orange region. The red “T” represents dislocations, the blue line represents subcrystalline boundaries, the white line represents crystal orientation, the area surrounded by the yellow curve represents disordered regions, and the orange area represents disordered domains. Copyright 2017 Wiley-VCH. (g) Photocatalytic performance of different ZIF-8 samples. (h) Comparison of the photocatalytic performance of a ZIF-8-1000 kGy and ZnO/ZIF-8 mixture. (i) Photocatalytic degradation of methyl bromide by ZIF-8-1000 kGy.^55,72^ Copyright 2022, Wiley-VCH.

As previously mentioned, ionizing irradiation can introduce defects into catalysts through ionization, electron excitation, and cascade collisions. Among these methods, ion irradiation is the most prominent because it allows for the precise control over the energy, type of incident ions, and position of the beam spot.^127^ This enables adjustment of the irradiation site, doping ion type, and number of defects on the catalysts, which is beneficial for photocatalysis and electrocatalysis. For example, Sun *et al.*^72^ reported an effective strategy for optimizing defects and interfaces in intermetallic PtPb nanosheets through 10 MeV C^+^ ion irradiation. The structure changed from single-crystal to polycrystalline, with the introduction of defects such as dislocations, subgranular boundaries, amorphization, and multiple defect-related interfaces (Fig. 9a–f). The optimized Pt nanoplates exhibited 2.97 times higher ORR specific activity and 3.00 times higher mass activity compared to the pristine Pt nanoplates. Additionally, they also exhibited excellent stability. This work emphasizes the superiority and significance of ion irradiation in inducing and tuning defects and interfaces for better performance. Kakitani *et al.*^128^ investigated the interfacial effects of Ar^+^-induced defects in glassy carbon substrates on the size and electronic structure of Pt NPs. First, a glassy carbon substrate was irradiated with Ar^+^ ions at 380 keV. Then, Pt nanoparticles were deposited on the substrate by radio frequency magnetron sputtering. The irradiation defects in the carbon substrate promoted the growth of Pt NPs on the substrate, resulting in larger Pt NPs. The DFT calculations revealed that lattice vacancies in the graphite structure lowered the position of the d-band centres, leading to higher ORR activity.

Devadoss *et al.*^129^ demonstrated that RGO films were significantly affected by γ-ray irradiation. The exfoliation and formation of defects on the surface of RGO nanosheets lead to additional edge sites and lots of hydrophilic groups. Meanwhile, oxygen doping into the GO lattice increases the hole size at the Fermi level, leading to the shift of the Fermi level towards positive potential, which is favorable for water oxidation. The enhanced hydrophilicity of 25 kGy C-irradiated RGO also increases hydroxide (OH^−^) adsorption and facilitates O_2_ desorption. As a result, at 1.0 V *vs.* Ag/AgCl, the ORR performance of the 25 kGy-GO catalyst was six times that of untreated RGO. Most recently, Rahman *et al.*^98^ synthesized N-doped rGO-supported iron catalysts with different iron loadings using γ-ray irradiation. The results show that γ-ray irradiation introduces both N and Fe into the samples while also generating structural defects. The strong interaction between Fe–N_*x*_ and O_2_ promotes rapid electron transfer. The ORR performance was further influenced by Fe loading, with higher Fe content introduced through irradiation leading to increased ORR activity.

### 4.2 Photocatalysis

Photocatalysis is a promising approach for producing low-cost hydrogen energy and decomposing organic pollutants.^21^ However, the limited number of active sites in photocatalysts creates a significant gap between the actual and theoretical efficiencies of photocatalysts. Thus, developing effective modification methods to enhance photocatalytic efficiency is essential to achieving optimal performance in photocatalytic hydrogen production and pollutant decomposition. Irradiation technology enables precise doping and defect control in photocatalysts, allowing for fine-tuning of their electronic structures and optimizing photocatalytic performance (Table 3).

**Table 3:** Summary of the application of various types of irradiation technology in synthesis/modification of photocatalysts from recent reports

Catalyst materials	Irradiation sources	Energy	Fluence	Roles	Application	Ref.
TiNRs, Ag-TiNRs, Au-TiNRs	Ion beam irradiation/P	50 keV	1 × 10^14^, 1 × 10^15^, 5 × 10^14^, 5 × 10^15^ ions per cm^2^	Defect engineering	Degradation of methylene blue	101
TiO_2_	γ-ray irradiation/^60^Co	1.2 MeV	7, 11 kGy	Band gap engineering	Degradation of methylene blue	130
ZIF-8@ZnO	Electron beam irradiation	1.5 MeV	50, 100, 250, 500, 750, 1000 kGy	Material synthesis and heterostructure formation	Degradation of methylene blue	55
TiO_2_/CNTs	Proton irradiation	120 keV	5 × 10^14^, 1 × 10^16^ cm^−2^	Defect introduction	Degradation of methylene blue	131
TiO_2_	Electron beam irradiation	6 MeV	5, 10, 20 kGy	Structural modification	Degradation of direct blue 1	132
MoS_2_	γ-ray irradiation/^60^Co	—	1, 10, 100, 1000 kGy	Band gap engineering	Degradation of methylene blue	133
Pb_0.98_Cu_0.01_Sr_0.01_S	γ-ray irradiation/^60^Co	25.2 MeV	90 kGy	Band gap engineering and defect engineering	Degradation of methylene blue	31
NiO	γ-ray irradiation/^60^Co	1.173, 1.332 MeV	180 Gy to 10 kGy	Phase transformation	Photocatalytic	83
WO_3_	Ion beam irradiation/He^2+^	40 keV	1 × 10^13^–10^15^ ions per cm^2^	Morphology control	Degradation of indigo carmine dye and Congo red	83
WO_3_	Ion beam irradiation/He^2+^	40 keV	1 × 1015 ion per cm^2^	Morphology control	Degradation of Rhodamine B	134
ZnO	Ion beam irradiation/N	100 keV	5 × 10^14^, 1 × 10^15^, 5 × 10^15^ ion per cm^2^	Band gap engineering and vacancy introduction	Degradation of methyl orange	135
In_2_O_3_:F	γ-ray irradiation/^60^Co	—	1, 5, 10, 100 kGy	Band gap engineering and defect engineering	Degradation of methylene blue	136
Ag/Bi_2_WO_6_/CdWO_4_	Electron beam irradiation	—	100 kGy	Defect engineering	Degradation of carmine, rhodamine B and Cr^6+^	76
rGO-TNTAs	γ-ray irradiation/^60^Co	—	0, 10, 20, 30, 40 kGy	Reduce GO to partial rGO	Degradation of ethylene (C_2_H_4_)	137
COF	Electron beam irradiation	1.5 MeV	5, 100, 300, 500 kGy	Material synthesis	Photoelectrochemical water splitting	64
WO_3_	Ion beam irradiation/Zr^+^	3 keV	0.33 × 10^15^, 1 × 10^15^, 3 × 10^15^ ions per cm^2^	Zr-doping and band gap engineering	Photoelectrochemical water splitting	138
TiO_2_	Ion beam irradiation/Ar^+^	190 keV	1 × 10^12^, 5 × 10^12^, 5 × 10^13^, 5 × 10^14^ ions per cm^2^	Defect engineering	Photoelectrochemical water splitting	139
TiO_2_ nanorods	Ion beam irradiation/N^+^	65 keV	1 × 10^17^ ions per cm^2^	Nanostructure formation	Photoelectrochemical water splitting	140
TiO_2_ nanotubes	Ion beam irradiation/N	60 keV	1 × 10^15^, 1 × 10^16^ ions per cm^2^	N-doping	Photoelectrochemical water splitting	99 and 141
ZnO nanorod arrays	Ion beam irradiation/Cu	30 keV	3 × 10^5^, 5 × 10^15^, 2 × 10^16^ ions per cm^2^	Doping	Photoelectrochemical water splitting	107
ZnO nanorod arrays	Ion beam irradiation/V	50 keV	2.5 × 10^14^–5 × 10^15^ ions per cm^2^	Doping	Photoelectrochemical water splitting	108
TiO_2_ nanowire arrays	Ion beam irradiation/N	30 keV	7 × 10^14^, 1 × 10^16^ ions per cm^2^	N-doping	Photoelectrochemical water splitting	142
ZnO nanorod arrays	Ion beam irradiation/N	30 keV	1.25 × 10^14^–5 × 10^15^ ions per cm^2^	N-doping	Photoelectrochemical water splitting	143
TiO_2_ nanowire arrays	Ion beam irradiation/C, N	25, 30 keV	1 × 10^15^, 1 × 10^16^ ions per cm^2^	Doping	Photoelectrochemical water splitting	144
α-Fe_2_O_3_	Ion beam irradiation/Au	30 keV	1 × 10^16^, 2.5 × 10^16^, 5 × 10^16^, 1 × 10^17^ ions per cm^2^	Heterostructure formation	Photoelectrochemical water splitting	145

#### 4.2.1 Photocatalytic degradation of organic pollutants

Photocatalytic degradation of organic pollutants produces highly active free radicals that react with or replace organic contaminants, offering an effective method for environmental remediation.^146^ It has many advantages, such as environmental protection, energy-saving and easy operation. However, the development of efficient photocatalysts with excellent activity and stability is crucial for the practical implementation of this technology in water and air purification. Recently, Wang *et al.*^55^ synthesized a ZIF-8@ZnO heterostructure by using electron beam irradiation and investigated its photocatalytic ability toward methylene blue (MB). Under simulated sunlight, ZIF-8-1000 kGy showed fast degradation kinetics and high degradation efficiency for MB, which was superior to those of the ZIF-8 prepared by a solvothermal method (Fig. 9g). In addition, a physical mixture of ZnO and ZIF-8 was also tested, and the corresponding degradation rate was significantly lower than that of ZIF-8-1000 kGy (Fig. 9h). The increased photocatalytic activity was attributed to the following reason: Firstly, the layered pore structure enables ZIF-8-1000 kGy to pre-enrich MB from solution, as demonstrated under dark conditions, which facilitates faster degradation when exposed to light (Fig. 9i).

Secondly, the formed ZIF-8@ZnO heterostructure facilitates photogenerated charge separation. These results demonstrate that electron beam irradiation is a promising method for compositing ZIF-8 and forming ZIF-8@ZnO heterostructures, offering significant advantages over conventional solvothermal reactions. Wang *et al.*^111^ fabricated CdTe/ZnO heterostructures by N ion irradiation. The photocatalytic activity was improved after N ion irradiation treatment due to the introduction of oxygen vacancy defects. Lv *et al.*^135^ fabricated ZnO nanowires on Si substrates *via* a hydrothermal growth method, followed by an N ion irradiation treatment with an energy of 100 keV (fluences of 5 × 10^14^, 1 ×10^15^, and 5 × 10^15^ ions per cm^2^). Compared with the as-grown ZnO nanowires, the photocatalytic performance of ZnO heterostructures was improved by the reduction of the band gap and the generation of oxygen vacancies at 5 × 10^15^ ions per cm^2^. The reduction of the band gap promotes the transfer of electrons and holes, which improves the charge separation efficiency and visible light absorption. On the other hand, the N ion radiation increases the gap of oxygen vacancies and hinders the recombination of photoexcited electrons and holes. Gallegos *et al.*^132^ investigated the degradation of Direct Blue 1 by using a 6 MeV electron beam irradiation of TiO_2_. The 10 kGy irradiation of TiO_2_ allows DB1 to degrade more rapidly than the degradation with TiO_2_ irradiated at 0, 5, and 20 kGy. The absorption rate of the TiO_2_ irradiated at 10 kGy is lower than that of the other used materials (TiO_2_ irradiated at 0, 5, and 20 kGy). This is obviously favourable to the degradation of DB1, as excessive adsorption would prevent sufficient light from passing through the photocatalyst, resulting in a reduction in the rate of degradation.

Bamola *et al.*^101^ irradiated TiO_2_ nanorod (TiNR)-based hybrids (Ag-TiNRs and Au-TiNRs) with varying fluences of P ions at 50 keV. They found that Ag-TiNRs irradiated at a fluence of 5 × 10^14^ ions/cm^2^ degraded 78% of methyl bromide under visible light irradiation, outperforming samples irradiated at 1 × 10^14^ ions per cm^2^ (65%), 1 × 10^15^ ions per cm^2^ (74%), and 5 × 10^15^ ions per cm^2^ (68%). Notably, the best degradation performance was achieved with Au-TiNRs irradiated at 5 × 10^14^ ions per cm^2^, achieving an 84% degradation rate, which was superior to that of samples irradiated at 1 × 10^14^ ions per cm^2^ (80%), 1 × 10^15^ ions per cm^2^ (76%), and 5 × 10^15^ ions per cm^2^ (73%). The study also indicated that these hybrid nanostructures are modified at low fluence, while high fluence leads to structural damage. Low-fluence ion implantation of Ag-TiNRs and Au-TiNRs reduces surface states and enhances charge-harvesting efficiency, with less structural damage compared to high fluence. As a result, charge carriers are more effectively separated in low-fluence hybrid materials, thereby improving photocatalytic activity.

As discussed earlier, e-beam irradiation has proven to be an effective method for surface modification and the induction of microstructural evolution in electrocatalysts. Notably, this technique also has potential applications in the field of photocatalysts.^147^ For example, Latthe *et al.*^148^ altered the micro/nanostructure of photocatalysts by electron beam treatment. The catalytic activity of the irradiated photocatalysts in water splitting was greatly improved. Similarly, Chen *et al.*^149,150^ also demonstrated that the oxygen containing groups in the GNSs could be partially removed by medium electron beam irradiation, leading to a significant improvement of the lithium storage performance. In related work, the same group irradiated SnS_2_ quantum dots (QDs)/amino-functionalized graphene nanosheets (SAGNSs) with electron beams at doses of 70, 140, and 280 kGy.^151^ They observed that N-doped graphene nanosheets (NGNSs) were successfully prepared by the e-beam irradiation, and the microstructure was further refined. Additionally, it allowed precise control over the crystallinity and lattice defects in SnS_2_ QDs, influencing the amorphization of NGNSs and the surface oxygen-containing groups. The performance of SAGNSs and SnS_2_ quantum dots/N-doped graphene nanocomposites (SNGNSs) for degradation of methyl orange (MO) was evaluated under the same experimental conditions. The results showed that the photodegradation rate of SNGNSs reached 95.6% under the irradiation condition of 70 kGy, and that of SAGNSs reached 59.5% after 60 min. The rate constants of SNGNSs were 3.19 times higher than those of SAGNSs at 70 kGy. These findings indicate that e-beam irradiation is a versatile and effective method for the surface modification of graphene-based nanocomposites, enabling the construction of photocatalysts with better performance.

Xie *et al.*^137^ utilized γ-ray irradiation to reduce rGO and investigated the photocatalytic properties of rGO-modified TiO_2_ nanotube arrays (rGO-TNTAs) for ethylene degradation under UV light. It was found that the degradation rate (*K*) increased first, then decreased, and finally reached the highest value when the dose was 20 kGy. This trend corresponded with a decrease in the ID/IG ratio from 0.9174 to 0.8468. The enhanced photocatalytic activity was attributed to a strong interaction between TiO_2_ and rGO, facilitated by defects in the rGO structure. After the 20 kGy γ-ray irradiation, optimal changes occurred in the D band to G band intensity ratio of GO, resulting in a 40.9% increase in K for rGO-TNTAs compared to TNTAs following 120 keV proton beam irradiation. Simultaneously, the CNTs experienced damage, a reduction in tube diameter, and a decrease in the Raman peak ID/IG ratio with increased proton fluence. Notably, the nano-TiO_2_/CNT films demonstrated remarkable MB degradation performance under irradiation. Furthermore, CNTs coated with thinner TiO_2_ layers exhibited relatively improved photocatalytic activity, likely due to the crystallization of the TiO_2_ layer and the carrier-trapping effect of the CNTs.

Recently, a research group led by Martinez D conducted a series of experiments to study the photocatalytic properties of WO_3_ microparticles in organic dyes. They elucidated the size dependence of photocatalytic activity, showing that smaller grain sizes and larger active surface areas corresponded to increased photoactivity and reaction rates.^152–155^ In related work, Kozovskiy *et al.*^156^ studied the effect of low-energy WO_3_ microparticle irradiation on the structure and photocatalytic activity of organic dyes. Their findings showed that an increase in radiation dose led to small but significant changes in surface area. At a dose of 10^13^ ions/ per m^2^, there was a 3.7% increase in Brunauer–Emmett–Teller (BET) surface area. Further dose increases to 10^14^ and 10^15^ ions per cm^2^ resulted in larger increases of 13.8% and 28.1% in BET surface area, respectively. Photocatalytic testing revealed that the initial, non-irradiated WO_3_ microparticles achieved only 45–50% degradation of indigo carmine dye after 300 min. However, complete degradation was observed after 200 min for microparticles irradiated at 10^13^ ions per cm^2^, and after 270 min for those irradiated at 10^14^ ions per cm^2^. Notably, partial amorphization at 10^15^ ions per cm^2^ led to a reduction in degradation and a decrease in photocatalytic activity. Similarly, in the decomposition of Congo red dye, non-irradiated WO_3_ microparticles degraded no more than 20% of the initial dye concentration. In contrast, irradiation-modified particles achieved 45–50% degradation. Kozovskiy *et al.*^134^ further explored the effects of irradiation on WO_3_ microparticles in additional experiments. The effect of irradiation of commercial WO_3_ microparticles with low energy He ions toward Rhodamine B degradation was studied. For the initial microparticles without irradiation, reduction in the maximum absorption intensity takes place and the intensity gradually reaches the minimum after 300 min of testing, whereas in the case of the modified microparticles, the change of the solution is observed after 210 min. The rate of photocatalytic decomposition proceeds much more quickly for the irradiated samples, with a reduction in the band gap value and a shift in the fundamental absorption edge, indicating an obvious change in the electron density of catalysts.

#### 4.2.2 Photoelectrochemical water splitting

Photoelectrochemical (PEC) water splitting is considered to be one of the most promising strategies for the generation of hydrogen. However, PEC water splitting is a complicated process, which involves several sequential physical and chemical reactions, including light-absorbing, photo-excited, and surface-oxidation-reduction reactions. The development of low cost, high efficiency, and stable semiconductor-based photocatalysts is a key step in the development of economically feasible PEC energy transformation worldwide. Despite there being hundreds of studies focused on various photocatalysts, TiO_2_ is still one of the most frequently used semiconductive materials. Nevertheless, when TiO_2_ is used as a catalyst without a co-catalyst such as Pt, Pd, or Au, a major shortcoming is that the charge-transfer reaction kinetics and therefore the H_2_ generation rate are very slow. Coincidentally, ion beam irradiation is a powerful tool for tailing the structure of photocatalysts. In particular, helium ion implantation can be used to create nanoporous structures in various metals, which has been systematically investigated by Prof. Peter B. Johnson and colleagues.^157,158^ Liu *et al.*^85^ proposed an effective method for fabricating porous TiO_2_ nanorod arrays (NRAs). Crystalline rutile TiO_2_ NRAs were grown on FTO substrates and subjected to He^+^ ion implantation at different energies (Samples 1A, 2A, and 3A correspond to ion irradiation energies of 30, 50, and 70 keV, respectively). Helium ion implantation has advantages in altering material structure due to its light element nature, which causes less damage to the target material and promotes bubble formation. By selecting the ion energy, fluence, and temperature, this method offers the possibility of controlling the pore size of photocatalysts. The TiO_2_ NRAs obtained after irradiation were found to be porous structures with internal nanocavities. The nanocavities formed in the nanorods trap holes and facilitate the separation of electron–hole pairs. The photocurrent densities increased to 0.25, 0.38, and 0.36 mA cm^−2^ at 0.5 V *vs.* SCE, respectively, compared to the pure TiO_2_ NRAs (0.19 mA cm^−2^).

Zhou *et al.*^159^ investigated the impact of N implantation on the structure of semiconducting TiO_2_ at 8 × 10^14^ ions per cm^2^ and 1 × 10^16^ ions per cm^2^ at 60 keV. At a low irradiation dose, it activated anatase TiO_2_ nanotubes for noble metal-free photocatalytic H_2_ generation. Low-dose nitrogen implantation increases the sub-bandgap response of the anatase, which reduces the charge-transfer resistance. However, high doses have an adverse effect on the photocurrent magnitude, leading to excessive charge transfer efficiency. The implantation of various elements in particulate photocatalysts has been shown to significantly enhance photocatalytic performance. Ion beam irradiation is also readily available for various doping modifications of photocatalysts. Additionally, ion implantation introduces oxygen vacancies that lead to an upward shift of the valence band maximum. These findings suggested that the upshift of the conduction and valence bands is beneficial for photoelectrochemical water splitting. Wang *et al.*^107^ successfully produced Cu-doped ZnO nanorod arrays under visible light using ion irradiation. The photocurrent density reached 18 μA cm^−2^ (with respect to the saturated mercuric glycol electrode), which is approximately 11 times higher than that of the undoped ZnO nanorod arrays (0.8 V).

**Fig. 10:**
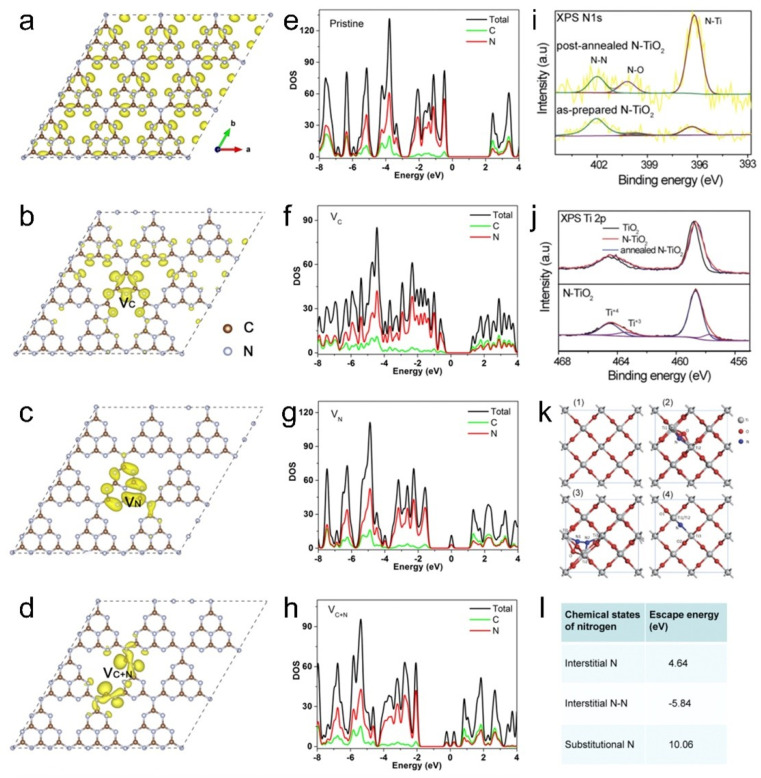
Structural models of g-C_3_N_4_: (a) pristine, (b) with carbon vacancies, (c) with nitrogen vacancies, and (d) with both carbon and nitrogen vacancies. (e–h) DOS of post-implantation annealed N–TiO_2_ and prepared N–TiO_2_. Copyright 2019, Wiley-VCH. (i) XPS spectra of N 1s. (j) Upper panel: XPS Ti 2p spectra for air-annealed pristine TiO_2_, as-prepared N-TiO_2_ and annealed N-TiO_2_. Lower panel: Ti 2p fitted curve for N–TiO_2_. (k) Relaxation structures of (1) pristine TiO_2_, (2) interstitial N-doped TiO_2_, (3) interstitial N-doped TiO_2_ and (4) substituted N-doped TiO_2_. (l) Calculated escape energies for various nitrogen chemical states.^142,164^ Copyright 2015, American Chemical Society.

Graphitic carbon nitride (g-C_3_N_4_) has been considered as an efficient metal-free catalyst for photocatalytic water splitting, but its performance is constrained by lower charge carrier separation efficiency and less visible light absorption. It has been reported that the visible light absorption and charge carrier separation efficiency of g-C_3_N_4_ can be enhanced by introducing carbon vacancies or nitrogen vacancies. There are several methods to produce N or C vacancies in g-C_3_N_4_, including hydrogen treatment, calcination in an Ar, N_2_, or NH_3_ atmosphere, and thermal polymerization at high temperatures or in strongly alkaline environments.^42,160–163^ However, the above common chemical methods can only produce one kind of vacancy (V_C_ or V_N_) and it is very difficult to control its concentration. In addition, these processes are complex and cumbersome and may introduce impurities. To solve this problem, Wang *et al.*^164^ have developed an ion beam irradiation method to fabricate defective g-C_3_N_4_ nanosheets with the annealing process. They found that the concentration and distribution of incident vacancies can be tuned by incident ion beam energy and fluence. During ion irradiation, vacancies of carbon and nitrogen are simultaneously introduced into g-C_3_N_4_ due to the cascade collisions between irradiated ions and target atoms. DFT calculations presented in Fig. 10a–h demonstrate that the introduction of V_C_ with irradiation leads to a narrowing of the bandgap, along with a shifted absorption edge. Local charge density (LDOS) analysis shows that electrons are more inclined around the V_N_, suggesting effective capture of photogenerated electrons by the V_N_. This defective state has the potential to improve the performance of photocatalysts and can be used as a high performance photocatalyst for PEC water decomposition.

**Fig. 11:**
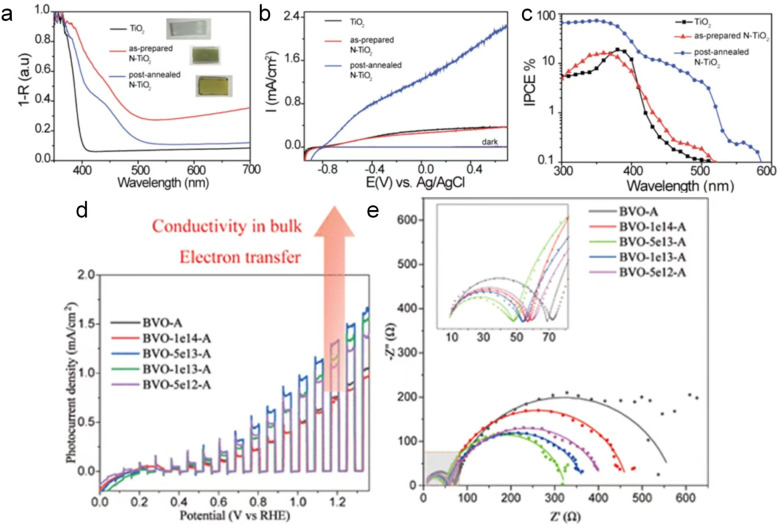
The PEC catalytic properties of TiO_2_, as-prepared N-TiO_2_ and annealed N-TiO_2_. (a) Photoabsorption properties, (b) linear swept voltammetry curves with a 100 mW cm^−2^ xenon lamp in a 1.0 M NaOH aqueous electrolyte, (c) IPCE spectra collected at 0.5 V *vs.* Ag/AgCl. The PEC catalytic properties of pristine Fe_2_O_3_, Au–Fe_2_O_3_-550, and Au–Fe_2_O_3_-700. Copyright 2015, American Chemical Society. (d) Mott–Schottky curves of BVO-A and the irradiated BiVO_4_ films in the dark. (e) EIS curves (inset: the equivalent circuit model) at 1.23 V *vs.* RHE under AM 1.5 G illumination.^145,165^ Copyright 2022, ACS Publications.

Wang *et al.*^142^ reported the enhancement in the performance of N–TiO_2_ for visible light-driven PEC water splitting through ion beam irradiation. As shown in Fig. 10i–l, the DFT calculations and XPS characterization revealed that the annealed substituted N dopants became the major introduced species. One can see that the annealed N^+^ implanted TiO_2_ exhibited an improved ability to absorb visible light. The optical band gap of the implanted N-TiO_2_ (2.34 eV) is significantly reduced compared to that of the pristine TiO_2_ (3.0 eV). On the other hand, the carrier injection efficiency is not significantly improved after N^+^ implantation, indicating that there is no obvious change in kinetics of the surface water oxidation. The results suggested that N^+^ ion injection improves the performance of the photoanode mainly by increasing the carrier separation efficiency. After the annealing process, the photocatalytic performance of N^+^ ion-incorporating TiO_2_ was improved. The photocurrent density of N-TiO_2_ after annealing was −1.92 mA cm^−2^ at 0.5 V *vs.* Ag/AgCl, which was 4 times higher than that of pure TiO_2_ nanowires and N^+^ implanted TiO_2_ nanowires without the annealing process (Fig. 11a–c). Remarkably, the incident photon-to-current efficiency (IPCE) of N^+^-implanted TiO_2_ at 450 nm reaches an impressive 17%, the highest reported among all studies on TiO_2_-based photoanodes for PEC water splitting without the addition of co-catalysts. However, excessive N^+^ fluence decreases the PEC performance and separation efficiency of photoexcited carriers at the optimal defect concentration. The samples were subsequently annealed.

Ion irradiation can be used to control the implanted elements, and multi-element doping can be achieved and multi-element synergistic modification can be realized. Song *et al.*^144^ reported for the first time the utilization of a C/N co-doping strategy to achieve the “Midas Touch” transformation of single-crystal linear rutile TiO_2_ NW arrays in visible-light PEC water separation. It was demonstrated that good synergistic interactions between C/N dopant atoms ensured a high concentration of substituted n-ti states and Ti^3+^ morphologies in the C/N co-doped TiO_2_ NW, which resulted in a significant enhancement of visible-light absorbance, charge separation and transfer efficiency. Therefore, the visible light photoactivity of C/N co-doped TiO_2_ nanocrystals is nearly 253 times higher than that of pure TiO_2_ nanocrystals. Moreover, C/N-TiO_2_ achieved high incident photon/electron conversion efficiency without using any other co-catalysts. The ion implantation doping combined with co-doping strategy has been demonstrated to increase the photoelectrochemical conversion and improve the PEC water separation performance of semiconductor photocatalysts. Wu *et al.*^144^ introduced oxygen vacancies into TiO_2_ by Ar^+^ ion irradiation, resulting in enhanced photocatalytic performance. They found that low concentrations of oxygen vacancies could form shallow donor energy levels in conduction bands, which can trap photogenerated electrons and release them into the conduction band. The oxygen vacancies generated through irradiation increased the carrier concentration and inhibited photogenerated carrier recombination, thereby improving the PEC water separation properties of TiO_2_.

Most recently, He *et al.*^145^ reported an effective route to improve the PEC water splitting properties of α-Fe_2_O_3_ by using Au ion irradiation followed by annealing. After annealing, Au atoms recrystallized, diffused and precipitated on the α-Fe_2_O_3_ nanorods to form Au nanoparticles, which promoted stronger contact between the formed Au particles and hematite, enhancing the surface charge injection (C–I) efficiency, which reached 89% at 1.5 V *vs.* RHE. Furthermore, the Au-implanted Fe_2_O_3_ photoelectrode exhibits high stability in a non-photocurrent decay test, with no noticeable photocurrent decay. This work suggests that heterogeneous structures, with embedded metal particles, can be readily formed on film surfaces through ion implantation and subsequent annealing, offering improved PEC performance.

Duan *et al.*^165^ fabricated nanoporous BiVO_4_ with abundant bulk oxygen vacancies through helium ion irradiation followed by vacuum annealing. Under optimal irradiation conditions, the PEC performance of the irradiated samples was significantly improved. As shown in Fig. 11d and e, with an ion flux of 5 × 10^13^ ions per cm^2^, the photocurrent density was significantly increased by about 70%, and the photonic current efficiency doubled compared to that of untreated BiVO_4_. The enhanced PEC performance is attributed to the formation of bulk oxygen vacancies, which improve conductivity, enhance electron–hole pair separation efficiency, and accelerate charge transfer. The results indicate that ion irradiation is a powerful tool to accurately tailor the electronic structure of BiVO_4_ by inducing oxygen vacancies, thus improving the PEC performance of the material. Moreover, this controllable technique can be further applied to other semiconductor photoelectrodes. The results provide strong evidence that ion beam technology can enhance PEC catalytic performance by manipulating the catalyst structure, implementing co-doping, and generating heterostructures. These findings underscore the importance of accurately controlling irradiation fluence to optimize PEC activity, offering valuable insights for developing advanced photocatalytic materials with tailored performance characteristics.

## 5 Future perspectives

In summary, this review provides a comprehensive overview of the characteristics of ionizing and non-ionizing radiation, highlighting their advantages over conventional methods for the efficient and rapid synthesis of photo-/electrocatalysts. Additionally, a detailed summary of recent advances in ionizing irradiation for renewable energy materials, particularly in electrocatalytic and photocatalytic applications, is presented. Ionizing radiation enables heteroatom-doping, defect engineering and heterostructure formation. Tables 2 and 3 provide a summary of irradiation applications in the modification and optimization of catalytic materials. Radiation technology has emerged as a promising and efficient tool with applications that can extend to various other fields.

(1) Some studies have demonstrated that irradiation technology, different from other chemical methods, can introduce defects within catalysts. Therefore, it becomes crucial to perform targeted simulation calculations of the irradiation effect to guide and predict various doping elements and defect control strategies. SRIM calculations have been employed to simulate the penetration depth of ion beam irradiation in materials. However, accurately determining the penetration depths of electron beams, proton beams, and gamma rays remains a challenge and often requires Monte Carlo simulation methods.

(2) Irradiation has proven to be a highly precise and advanced technique for catalyst modification. For example, it enables the determination of the structure–activity relationship between defect concentration and catalytic properties. However, the underlying mechanisms of the interaction between irradiation processes and structural changes in catalysts require further investigation. The use of *in situ* characterization technology, such as *in situ* TEM and Raman measurements, can provide valuable insights into the continuous atomic structural changes in catalyst materials and their intrinsic relationship with the evolution of catalytic properties.

(3) Currently, the modification of catalysts by irradiation primarily relies on a single type of irradiation. This, while effective, may have limitations in fully optimizing catalytic performance for specific applications. By strategically choosing different irradiation types and combining them synergistically, more approaches could be achieved. This will open up the potential to develop novel catalysts with better performance, allowing for applications in new scenarios. For example, such advancements could address critical processes like carbon dioxide reduction and nitrogen fixation, providing pathways for the creation of advanced materials that meet the evolving demands of diverse catalytic applications and align with sustainability goals.

(4) At present, the modification of catalysts through irradiation primarily revolves around precious metal-based materials, due to their unique catalytic properties and broad applications. The continuous improvements in equipment and characterization techniques are paving the way for innovative approaches such as the development of single-atom catalysts. It holds the potential to embrace various catalyst types with tailored structures and enhanced performances, which may revolutionize diverse catalytical applications and contribute to sustainable advancements in materials science.

(5) With the continuous development of various accelerator technologies, the utilization of proton irradiation and neutron irradiation will play an important role in practical industrial production. The high throughput and cost-effectiveness of irradiation technology make it an attractive option for large-scale applications. Its unique ability to modify catalysts at multiple levels, including atomic and molecular levels, presents vast opportunities for improving product quality and reducing costs. This transformative capability contributes significantly to advances in efficiency, sustainability, and innovation across various sectors. The impact of these advanced irradiation techniques on catalysis production needs further exploration and in-depth research. Understanding how these techniques can be harnessed to enhance the catalytic performance of catalysts will undoubtedly uncover new possibilities, driving the evolution of industrial catalysis and paving the way for more sustainable and efficient production methods.

## Conflicts of interest

There are no conflicts to declare.
